# The aerial epidermis is a major site of quinolizidine alkaloid biosynthesis in narrow‐leafed lupin

**DOI:** 10.1111/nph.20384

**Published:** 2025-01-14

**Authors:** Karen Michiko Frick, Marcus Daniel Brandbjerg Bohn Lorensen, Nikola Micic, Eddi Esteban, Asher Pasha, Alexander Schulz, Nicholas James Provart, Hussam Hassan Nour‐Eldin, Nanna Bjarnholt, Christian Janfelt, Fernando Geu‐Flores

**Affiliations:** ^1^ Section for Plant Biochemistry and Copenhagen Plant Science Centre, Department of Plant and Environmental Sciences University of Copenhagen 1871 Frederiksberg Denmark; ^2^ Department of Pharmacy University of Copenhagen 2100 Copenhagen Denmark; ^3^ Department of Cell and Systems Biology/Centre for the Analysis of Genome Evolution and Function University of Toronto Toronto ON M5S 3G5 Canada; ^4^ Section for Transport Biology, Department of Plant and Environmental Sciences University of Copenhagen 1871 Frederiksberg Denmark; ^5^ Section for Molecular Plant Biology, Department of Plant and Environmental Sciences University of Copenhagen 1871 Frederiksberg Denmark

**Keywords:** laser‐capture microdissection, LCM, lupin alkaloids, *Lupinus angustifolius*, MALDI‐MSI, plant specialized metabolites

## Abstract

- Lupins are promising protein crops that accumulate toxic quinolizidine alkaloids (QAs) in the seeds, complicating their end‐use. QAs are synthesized in green organs (leaves, stems, and pods) and a subset of them is transported to the seeds during fruit development. The exact sites of biosynthesis and accumulation remain unknown; however, mesophyll cells have been proposed as sources, and epidermal cells as sinks.
- We investigated the exact sites of QA biosynthesis and accumulation in biosynthetic organs of narrow‐leafed lupin (*Lupinus angustifolius*) using mass spectrometry‐based imaging (MSI), laser‐capture microdissection coupled to RNA‐Seq, and precursor feeding studies coupled to LC‐MS and MSI.
- We found that the QAs that accumulate in seeds (‘core’ QAs) were evenly distributed across tissues; however, their esterified versions accumulated primarily in the epidermis. Surprisingly, RNA‐Seq revealed strong biosynthetic gene expression in the epidermis, which was confirmed in leaves by quantitative real‐time polymerase chain reaction. Finally, feeding studies using a stably labeled precursor showed that the lower leaf epidermis is highly biosynthetic.
- Our results indicate that the epidermis is a major site of QA biosynthesis in narrow‐leafed lupin, challenging the current assumptions. Our work has direct implications for the elucidation of the QA biosynthesis pathway and the long‐distance transport network from source to seed.

## Introduction

Plants produce an immense array of specialized metabolites with important applications in the agricultural, pharmaceutical, and food industries. The biosynthesis and accumulation of plant specialized metabolites are often under strict control, being synthesized in specific cell‐types/tissues and often stored in others. For example, in the Solanaceae family, the first steps of tropane alkaloid biosynthesis occur in the root pericycle; intermediate steps occur in the root endodermis and outer cortex; and final steps once again occur in the root pericycle (Nakajima & Hashimoto, 1999; Suzuki *et al*., 1999a,b). The tropane alkaloids are then transported from the roots to the aerial parts where they are stored. In turn, the biosynthesis of monoterpene indole alkaloids (MIAs) in leaves/stems of *Catharanthus roseus* involves at least three types of cells: early steps occur in the internal phloem‐associated parenchyma, intermediate steps take place in epidermal cells, and final steps occur in idioblast or laticifer cells, where most of the MIAs also localize (St‐Pierre *et al*., 1999; Yamamoto *et al*., 2016). A thorough understanding of the biosynthesis and accumulation of plant specialized metabolites is crucial for understanding their function and for altering their production and/or accumulation in native or heterologous hosts.

Quinolizidine alkaloids (QAs) are a class of toxic specialized metabolites found in lupins (*Lupinus* spp.) and other Genistoid legumes. Four lupin species are currently cultivated for their high‐protein seeds: narrow‐leafed lupin (NLL; *L. angustifolius*), white lupin (*L. albus*), yellow lupin (*L. luteus*), and Andean lupin (*L. mutabilis*). Of these, NLL is the most widely cultivated (Vishnyakova *et al*., 2021). QAs can accumulate to high levels in the seeds (up to *c*. 3%), and even the low‐QA varieties (‘sweet’ varieties) often exceed the industry threshold to be used as food and feed (0.02% in Australia and New Zealand (Schedule 19, 2015)). Interestingly, it has been shown that the QAs in the seeds are not made *in situ*, but are transported there from maternal tissues (Otterbach *et al*., 2019), likely via the phloem (Lee *et al*., 2006). However, it is not well understood where QAs are made in the maternal tissues, and neither is the long‐distance QA transport pathway from source to seed.

Studies performed in the 1980s provided foundational knowledge on where QAs are made and accumulated in lupin plants. The first enzyme of the QA biosynthesis pathway, lysine decarboxylase (LDC) was found to localize to the chloroplast stroma in *L. polyphyllus* leaves (Wink & Hartmann, 1982). Examination of the distribution of QAs in *L. polyphyllus* petiole sections using laser desorption mass spectrometry (LAMMA 1000) showed that the QA lupanine was only detected in the epidermis and the neighboring 1–2 subepidermal layers (Wink *et al*., 1984). To find out in which tissues QAs are made, a radiolabeled QA intermediate (cadaverine) was fed to isolated petiole epidermis, to the corresponding ‘mesophyll’ (petiole without the epidermis), and to intact petioles of *L. polyphyllus*. Incorporation of the intermediate into lupanine was detected in the ‘mesophyll’ and intact petioles, but not in the isolated epidermis (Wink & Mende, 1987). The authors noted that epidermal cells are usually devoid of chloroplasts, and thus, they hypothesized that QAs are made in chloroplast‐containing mesophyll cells. It was also hypothesized that QAs are transported from mesophyll to epidermal cells, which is consistent with the observed epidermal localization of lupanine as well as the high capacity of the isolated epidermis to take up externally supplied lupanine (Wink & Mende, 1987).

Decades later, the gene encoding LDC was identified in NLL (Bunsupa *et al*., 2012), and so was the gene encoding the second enzyme in the pathway, copper amine oxidase (CAO) (Yang *et al*., 2017). The reactions performed by LDC and CAO are shown as Supporting Information Fig. S1 along with an overview of the rest of the QA pathway, most of which remains uncharacterized (Mancinotti *et al*., 2022). Examination of *LDC* and *CAO* expression in whole NLL organs revealed that they are highly expressed in green parts of the plant – leaves, stems, and developing pods (Bunsupa *et al*., 2012; Yang *et al*., 2017; Frick *et al*., 2018). As expected, fluorescently tagged LDC was found to localize to plastids (Bunsupa *et al*., 2012). These studies seem to support the hypothesis that QAs are made in the ‘mesophyll’, or rather, in the chloroplast‐containing parenchyma of the above‐mentioned organs. It follows that QAs are transported from there to the epidermis.

Here, we set out to determine the exact sites of QA accumulation and biosynthesis in the QA‐producing organs of NLL (leaf, stem, and developing pod). In our studies, we used mass spectrometry‐based imaging (MSI), laser‐capture microdissection coupled to RNA‐Seq, and precursor feeding studies coupled to LC‐MS and MSI. Our results indicate that the epidermis (and not the mesophyll) is the major site of biosynthesis, leading to a new spatial model for QA biosynthesis and transport.

## Materials and Methods

### Plant growth conditions

Seeds of narrow‐leafed lupin (NLL, *L. angustifolius* L.) cv Oskar (a high‐QA cultivar obtained from Hodowla Roślin Smolice, Poland) were sown in 16 cm‐wide, 20 cm‐deep pots at a density of two seeds per pot in potting mix (Pindstrup Færdigblandig 2; www.pindstrup.dk). The plants were grown in a growth chamber at temperatures of 20 : 18°C (day:night), a photoperiod of 16 : 8 h (light : dark), and 60% relative humidity. Light was supplied at 220 μmol m^−2^ s^−1^ during the day.

### Preparation of tissue for cryosectioning

NLL tissues (leaf, stem, and whole pods with seeds) were collected from plants when the first pods reached 30 d after anthesis (DAA). The tissue was quickly cut into 1.5–3.0 cm length pieces, frozen in liquid nitrogen, and stored at −80°C until further analysis. Frozen tissue was freeze‐embedded as described before (Kawamoto & Kawamoto, 2021), but without tissue fixation. Briefly, embedding containers obtained from SECTION‐LAB Co. Ltd, Japan (www.section‐lab.jp) were filled with 2.5% (w/v) aqueous carboxymethyl cellulose (CMC) solution. The filled containers were pre‐chilled by partially submerging in a mixture of hexane and dry ice. Frozen tissue was placed and orientated in the CMC and the whole block was fully submerged in the hexane/dry ice mixture to completely freeze it without the tissue thawing. Embedded blocks were stored at −80°C. Leaf and stem tissues were embedded in a 2.0 × 1.5 cm container, while pod tissue was embedded in a 3.5 × 2.5 cm container.

### Metabolite imaging by MALDI‐MSI

#### Sectioning and freeze‐drying

CMC‐embedded leaf, stem, and pod tissues were transversely sectioned in a Leica CM3050S Cryostat at −30°C using Kawamoto's film method (Kawamoto & Kawamoto, 2021) adapted for plant samples (Montini *et al*., 2020) to obtain 12–14 μm sections. Briefly, adhesive cryofilm (type 3C [16UF] 2 cm; SECTION‐LAB Co. Ltd) was used to obtain intact sections, which were then mounted onto glass slides using double‐sided carbon tape (SPI supplies; 2spi.com). The sections were freeze‐dried under vacuum overnight.

#### Matrix deposition

2,5‐dihydroxybenzoic acid (DHB) matrix was sublimated on dried leaf and stem sections, while for dried pod sections it was applied by spraying. Sublimation was performed on a custom‐built sublimator at a temperature of 140°C, and spraying deposition was performed by spraying 300 μl of a 30 mg ml^−1^ DHB solution in methanol/water (90 : 10) using a custom‐built matrix sprayer, as described in detail elsewhere (Wenande *et al*., 2017). For the third replicate of leaf and pod tissues as well as the last two time points upon feeding with labeled precursor (18 and 24 h), 7.5 mg ml^−1^ DHB solution in ethanol/water/trifluoroacetic acid (90 : 10 : 0.1) was sprayed using HTX TM‐Sprayer (HTX Technologies, Chapel Hill, NC, USA), at 0.1 ml min^−1^ flow rate and nozzle temperature of 60°C. The total number of passes was 20, done in a horizontal movement pattern (HH), with line spacing of 2 mm and without drying time between passes. The nozzle velocity was set to 1250 mm min^−1^ and nozzle height to 40 mm. Gas flow was set to 3 l min^−1^ with gas pressure kept at 10 psi.

#### MS imaging

After deposition of the matrix, the samples were imaged on a Thermo QExactive Orbitrap mass spectrometer, equipped with an AP‐SMALDI5 ion source (TransMIT GmbH, Giessen, Germany). Imaging was performed in positive ion mode using a scan range of *m*/*z* 150–1050. For the third replicate of leaf and pod tissues as well as the last two time points upon feeding with labeled precursor (18 and 24 h) we used a timsToF‐fleX MALDI‐2 instrument (Bruker Daltonics, Billerica, MA, USA) with post‐ionization applied (MALDI‐2). The imaging was performed with 10 μm spatial resolution for leaf and 50 μm spatial resolution for the pod sample in positive mode, with a scan range of *m*/*z* 50–1050. A total of 50 shots per pixel were applied, with a laser frequency of 1 kHz, trigger delay of 10 μs for the MALDI‐2 postionization, and ‘Single M2’ laser geometry with active beam scan.

QAs were identified based on the accurate *m*/*z* ratios of their protonated forms in accordance to previous assignment in NLL plant organs using LC‐MS (Otterbach *et al*., 2019; Mancinotti *et al*., 2021). Phosphatidylcholine (34 : 2)—a component of cell membranes – was chosen as a control metabolite for imaging, which could be visualized across the whole area of the tissue sections. The complete list of *m*/*z* values for the QAs, L‐lysine, their isotopically labeled counterparts, and phosphatidylcholine (34 : 2) is available as Table S1. For data recorded using the AP‐SMALDI5‐Orbitrap, images were generated using the software, MSiReader 1.01 (Robichaud *et al*., 2013; Bokhart *et al*., 2017). Images were normalized by the total ion current (TIC) and the color scales were adjusted to best display the compounds of interest. For data recorded using the timsToF‐fleX MALDI‐2, images were generated using SciLS Lab v.2024a Pro software (Bruker, Billerica, MA, USA). Data were normalized using Root Mean Square (RMS) normalization and images were generated using the exact mass ± 0.01 Da error interval with RMS normalization applied.

### Tissue‐specific transcriptome analysis by LCM‐RNA‐Seq

CMC‐embedded leaf, stem, and pod tissues were sectioned under the same conditions as for MALDI‐MSI (see section above), but without the use of cryofilm, and this time obtaining 14–20 μm sections. Whilst still at −30°C, the CMC embedding media was carefully removed from the sectioned tissue as much as possible. The sections were thaw‐mounted onto SuperFrost Plus Slides (VWR; treated with RNAseZAP and dried) by very briefly warming the slide with the hand to thaw the section and immediately re‐freezing it on the cold inductive plate inside the cryostat (tissue was thawed for maximum 1–2 s). Mounted sections were freeze‐dried under vacuum overnight.

Laser‐capture microdissection (LCM) was carried out on a PALM‐Microbeam instrument (Zeiss) at room temperature. AutoLPC setting was used to manually collect cells (energy = 100, focus = 80). Whenever target cells were in close proximity to other cell types, the cut setting (energy = 100, focus = 80) was used before the AutoLPC capture of cells to remove the undesired cell types. Cells were collected in a 0.2 ml PCR tube cap filled with 25 μl RNAlater (Thermo Fisher Scientific, Waltham, MA, USA), for a maximum time of 45 min. Immediately after collection, the first step of RNA extraction was carried out by adding 100 μl of extraction buffer from the Arcturus PicoPure RNA Isolation Kit (Thermo Fisher Scientific) and incubating samples for 30 min at 42°C. Cell extracts were stored at −80°C before completing the rest of the PicoPure RNA isolation protocol, adjusting for the modified volume of cell extract. The quality and quantity of RNA was determined using an Agilent RNA 6000 Pico Kit (Agilent Technologies, Santa Clara, CA, USA). For each cell sample type, RNA from several extractions was pooled together until 10 ng was obtained for one sample. Due to the laborious nature of collecting cells using LCM, two biological replicates of each sample type were collected (using tissue collected from separate plants). For all RNA samples collected from pods, RIN values were > 7. For RNA samples collected from leaves, RIN values were > 6.5. For RNA samples collected from stems, RIN values were > 7, except for parenchyma samples (5.5 and 5.7) and one phloem sample (6.5). Traces obtained using the Agilent RNA 6000 Pico Kit are provided in Fig. S2. RNA samples were provided to Macrogen (South Korea) for library construction and sequencing. Libraries were prepared using SMART‐Seq™ Ultra™ Low Input RNA kit and sequenced on an Illumina platform (HiSeq X) to generate 151‐bp paired‐end reads.

Raw Illumina reads were pre‐processed using rCorrector (Song & Florea, 2015), and TRIMGalore! (v.0.6.4) to trim adapter sequences and low‐quality bases (< 5). Any reads mapping to the SILVA small and large subunit ribosomal RNA database (for Archaeplastida; release 138.1), were removed, as well as overrepresented sequences as determined by FastQc (v.0.11.9). Transcript expression of processed RNA‐Seq data was quantified using Salmon (Patro *et al*., 2017) with the coding sequences from the previously published transcriptome of NLL cv Oskar (Yang *et al*., 2017; BioProject ID: PRJNA494078) as an index. Transcripts per million (TPM) values were cross‐sample normalized between all samples (TMM normalization). Differential gene expression analysis was performed using DESeq2 (Love *et al*., 2014).

### Generation of a NLL eFP Browser

The eFP Browser framework described in Winter *et al*. (2007) was modified to accept the NLL gene identifiers from the previously published transcriptome of NLL cv Oskar (Yang *et al*., 2017). Previously obtained RNA‐Seq data (Yang *et al*., 2017) and our new LCM‐RNA‐Seq data were databased on the Bio‐Analytic Resource for Plant Biology (BAR) server at bar.utoronto.ca (Toufighi *et al*., 2005). Images representing the samples from which RNA was isolated (whole plant organs as well as microdissected leaf, stem and pod tissues) were generated using GIMP (www.gimp.org). XML files linking the appropriate regions of the images to their respective database samples were manually created in a text editor and were loaded into the Lupin eFP Browser (available at https://bar.utoronto.ca/efp_lupin/cgi‐bin/efpWeb.cgi).

### Analysis of QA biosynthetic gene expression by quantitative polymerase chain reaction

Leaf abaxial epidermis was peeled from fresh NLL leaves, similarly to the method described by Weyers & Travis (1981). Peeled tissue was frozen immediately in liquid nitrogen and stored at −80°C. The remaining leaf tissue (without the abaxial epidermis) was also collected. RNA was extracted from *c*. 30 mg of abaxial epidermis or the remaining leaf tissue using the Spectrum Plant Total RNA kit (Sigma‐Aldrich) including on‐column DNase I digestion (Sigma‐Aldrich). cDNA synthesis and quantitative polymerase chain reaction to determine the expression of *LDC* and *CAO* was carried out as described previously (Mancinotti *et al*., 2021).

### Confocal microscopy imaging of chloroplasts

At *c*. 30 DAA, leaf, stem, and pod tissues were dissected into thin sections including the epidermis, and mounted with perfluorodecalin for observation by a SP5‐X confocal laser scanning microscope equipped with a DM6000 microscope (Leica Microsystems, Wetzlar, Germany). Chlorophyll autofluorescence was visualized using excitation/emission wavelengths of 496/661–750. Images were subsequently acquired and processed using the microscope imaging software Leica Application Suite X (v.3.7.5.24914). Each sample type was imaged with three biological replicates, with similar results between the replicates (replicate images not shown). Z‐stack images were also collected to visualize chloroplasts in the epidermis and underlying cell layers.

### Feeding of detached leaves with isotopically labeled L‐lysine

Leaves of 4–6 wk old NLL plants were detached by cutting at the petiole, and detached leaves were fed continuously through the petiole with an aqueous solution of either 5 mM isotopically labeled L‐lysine (L‐lysine∙2HCl [^13^C_6_, ^15^N_2_], Cambridge Isotope Laboratories, Inc.) or 5 mM unlabeled L‐lysine (Sigma‐Aldrich). Leaves were fed in a growth chamber at the above‐mentioned conditions (‘Plant growth conditions’ in the Materials and Methods section) for 0, 6, 10, 18, and 24 h. Due to the day–night cycle in the growth chamber and the timing of the experiment, leaves fed for up to and including 10 h were subjected to uninterrupted light, whereas leaves fed for 18 and 24 h experienced a night cycle. Leaf tissues were harvested at each of the mentioned time points. First, the abaxial epidermis was peeled and collected. Some of the remaining leaf tissue without abaxial epidermis was also collected, and some was used to harvest the mesophyll. For the latter, remaining leaf tissue was attached to plastic slides with the adaxial epidermis facing down using a minimal amount of CMC embedding media. After freezing on dry ice, a scalpel blade was used to scrape off and collect mesophyll cells, carefully avoiding the adaxial epidermis. After harvesting, all tissues were immediately submerged in liquid nitrogen and stored at −80°C until further analysis.

### Analysis of fed leaves by LC‐MS

Tissue was pulverized with a steel ball homogenizer and metabolites were extracted from the powder using 100 μl extraction solvent (60% methanol in water, 0.06% formic acid, and 5 ppm caffeine as internal standard) per 3 mg of tissue. The mixtures were shaken vigorously for 90 min at room temperature after which they were centrifuged at maximum speed for 1 min. The supernatant was collected and diluted 1 : 5 with water. Each diluted extract was passed through a 0.22 μm filter and transferred to a glass vial for LC‐MS analysis.

LC‐MS analysis was performed on a Dionex UltiMate 3000 Quaternary Rapid Separation UHPLC+ focused system (Thermo Fisher Scientific). Separation was achieved on a Kinetex® 1.7‐μm C18 column (100 × 2.1 mm, 1.7 μm, 100 Å; Phenomenex, Torrance, CA, USA), including a SecurityGuard™ C18 guard column (2.1 mm, 2 μm; Phenomenex). Mobile phases A and B consisted of, respectively, 0.05% formic acid in water and 0.05% formic acid in acetonitrile. Analytes were eluted using the following gradient at a constant flow rate of 0.3 ml min: 0–0.5 min, 2% B (constant); 0.5–2.375 min, 2–6% B (linear); 2.375–7 min, 6–25% B (linear), 7–13 min, 25–100% B (linear); 13–14 min, 100% B (constant); 14–14.5 min, 100–2% B (linear); and 14.5–20 min, 2% B (constant). The injection volume of all samples was 2 μl. The UHPLC was coupled to a Compact micrOTOF‐Q mass spectrometer (Bruker, Bremen, Germany) equipped with an electrospray ion source (ESI) operated in positive mode. Mass spectrometer conditions were as described before (Otterbach *et al*., 2019; Mancinotti *et al*., 2021).

The complete list of *m*/*z* values for the target compounds and their isotopically labeled counterparts is available as Table S1. Labeled and unlabeled QAs were quantified based on the relative abundance of their protonated molecular ions (peak areas from extracted ion chromatograms ±0.005 Da) compared to the internal standard caffeine. L‐lysine was found to deaminate readily under analysis conditions; therefore, the relative abundance of both labeled and unlabeled L‐lysine was calculated based on the deaminated fragment (Table S1). At each time point, tissues from three fed leaves were analyzed (biological replicates). For statistical comparisons, data that did not follow a normal distribution was log‐transformed. For data following a normal distribution (before or after log‐transformation), one‐way ANOVA was used to assess statistical differences between the relative abundance of detected compounds between tissues (independently for each time point). A significant one‐way ANOVA was followed up by a Tukey test for multiple comparisons (95% confidence interval). For data that did not follow a normal distribution after log‐transformation, a Kruskal–Wallis test was used to determine statistical differences. A significant Kruskal–Wallis test was followed up by a Dunn's test for multiple comparisons. A single replicate for tissue collected from leaves fed with unlabeled L‐lysine was also analyzed to ensure there was no background signal for the *m*/*z* values of the isotopically labeled QAs.

## Results

### Localization of QAs in biosynthetic organs by mass spectrometry‐based imaging

To determine the localization of QAs in biosynthetic organs of NLL, we prepared cross sections of leaves, stems, and developing pods, and analyzed them via high‐resolution matrix‐assisted laser desorption/ionization‐MSI (MALDI‐MSI). We examined the distribution of eight QAs, including core QAs representing unmodified or minimally modified QA backbones (lupanine, 13‐hydroxylupanine, and angustifoline) as well as esterified QAs representing larger modifications resulting from conjugation to CoA‐activated acids (e.g. 13‐*cis*/*trans*‐cinnamoyloxylupanine) (see structures in Fig. S1). The results are described in the following three subsections below. In addition, images showing the distribution of the control membrane component phosphatidylcholine 34 : 2 are provided in Fig. S3. Furthermore, the mass spectra of selected pixels of the MALDI‐MSI data are provided in Fig. S4. All MALDI‐MSI data presented here has been uploaded to METASPACE, where it can be browsed online (https://metaspace2020.eu/project/lupinus_angustifolius_Frick‐et‐al). These MALDI‐MSI experiments were repeated twice, giving a total of three replicates with similar results (all replicates uploaded to METASPACE).

#### Leaf

**Fig. 1:**
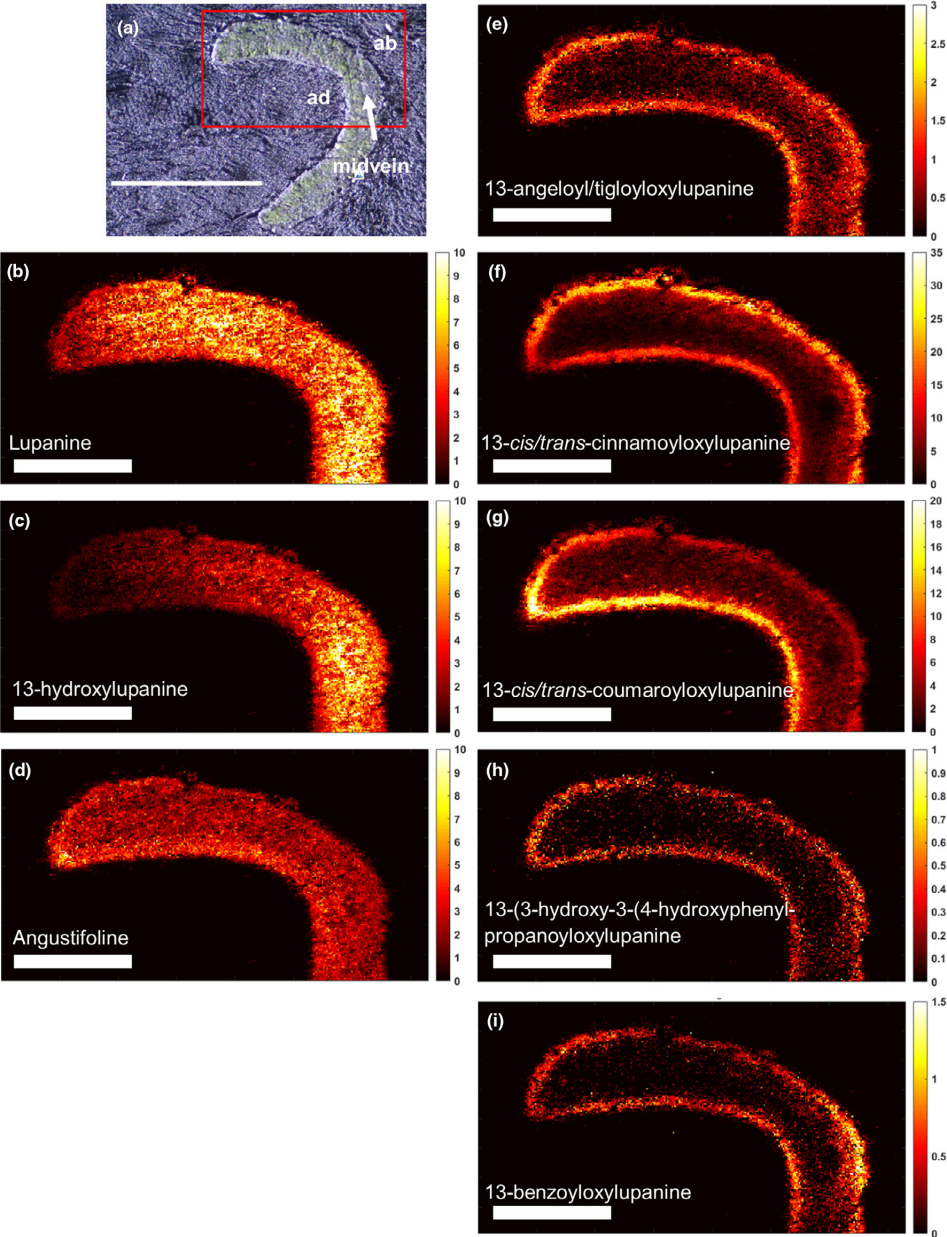
Transverse distribution of quinolizidine alkaloids (QAs) in a leaf of narrow‐leafed lupin (*Lupinus angustifolius*) as determined by matrix‐assisted laser desorption ionization mass spectrometry imaging (MALDI‐MSI). (a) Bright‐field microscopy image, with the red box denoting the area that was further analyzed by high‐res MALDI‐MSI (bar, 1 mm). ad, leaf adaxial (upper) surface; ab, leaf abaxial (lower) surface; midvein, central midvein. (b–i) Individual MALDI‐MS images of eight QAs at a spatial resolution of 5 μm (bar, 0.4 mm). Each image was obtained by selecting the exact mass of the protonated QA (±5 ppm, Supporting Information Table S1) and normalizing by the total ion current. The color scale represents signal intensity.

As the NLL leaf is bilaterally symmetrical, we imaged one half of the leaf including the central midvein (Fig. 1a). The core QAs lupanine and angustifoline appeared generally evenly distributed throughout the leaf, while 13‐hydroxylupanine appeared to increase in abundance towards the midvein (Fig. 1b–d). Interestingly, the esterified QAs showed a different distribution pattern and appeared to be most highly localized in the epidermis (Fig. 1e–i). An example of the replication of these results is presented in Fig. S5.

#### Stem

**Fig. 2:**
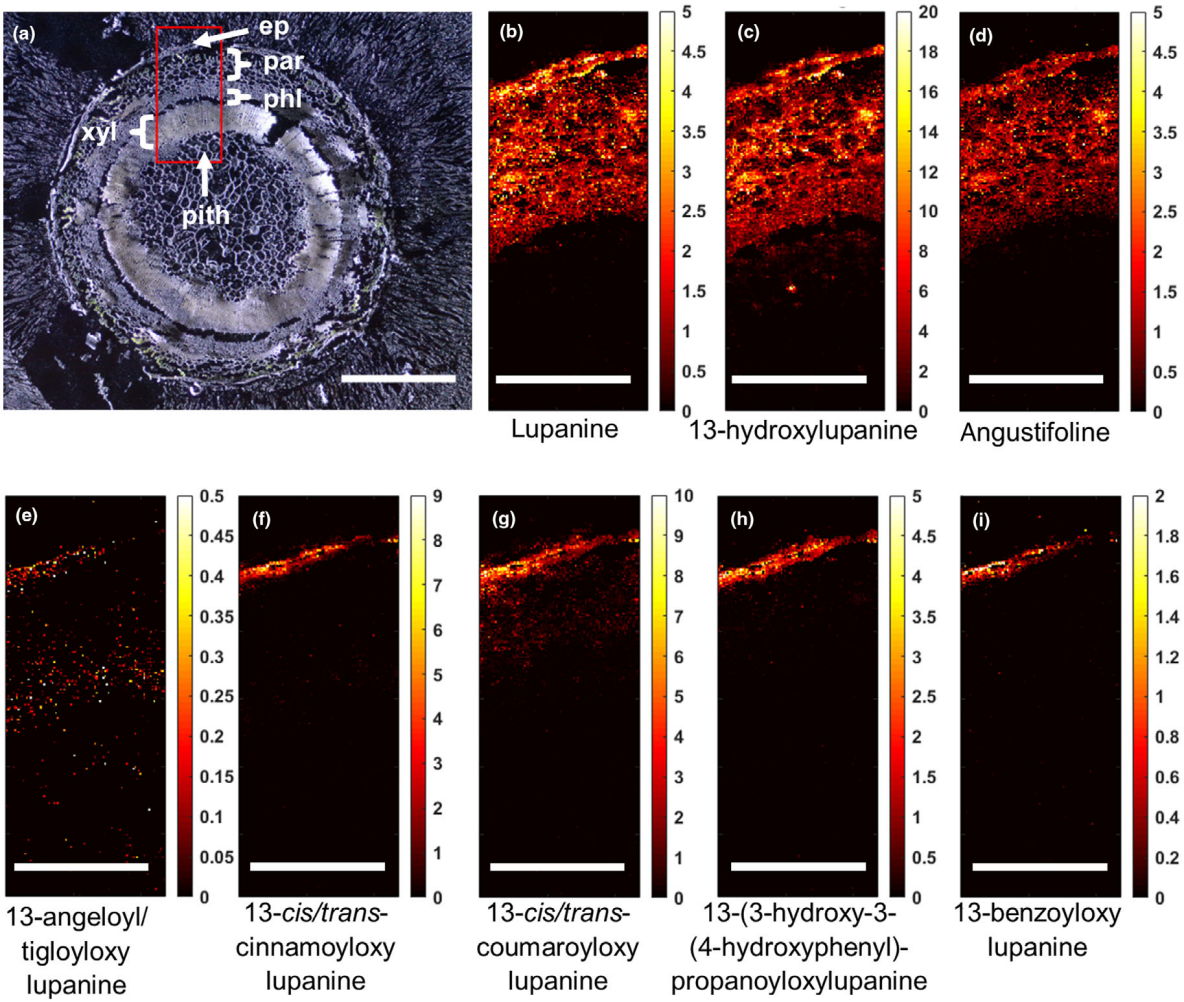
Transverse distribution of quinolizidine alkaloids (QAs) in a stem of narrow‐leafed lupin (Lupinus angustifolius) as determined by matrix‐assisted laser desorption ionization mass spectrometry imaging (MALDI‐MSI). (a) Bright‐field microscopy image, with the red box denoting the area that was further analyzed by high‐res MALDI‐MSI (bar, 1 mm). ep, epidermis; par, parenchyma; phl, phloem; xyl, xylem; pith, inner pith. (b–i) Individual MALDI‐MS images of eight QAs at spatial resolution of 5 μm (bar, 0.4 mm). Each image was obtained by selecting the exact mass of the protonated QA (±5 ppm, Supporting Information Table S1) and normalizing by the total ion current. The color scale represents signal intensity.

As the stem is radially symmetrical, we imaged a representative section containing all cell types from the outer epidermis to the inner pith (Fig. 2a). The core QAs were generally evenly distributed throughout the outer stem tissue, up to and including the phloem, but were not detected in the xylem or inner pith (Fig. 2b–d). The esterified QAs seemed to be localized in the epidermis (Fig. 2e–i).

#### Pod with seed

**Fig. 3:**
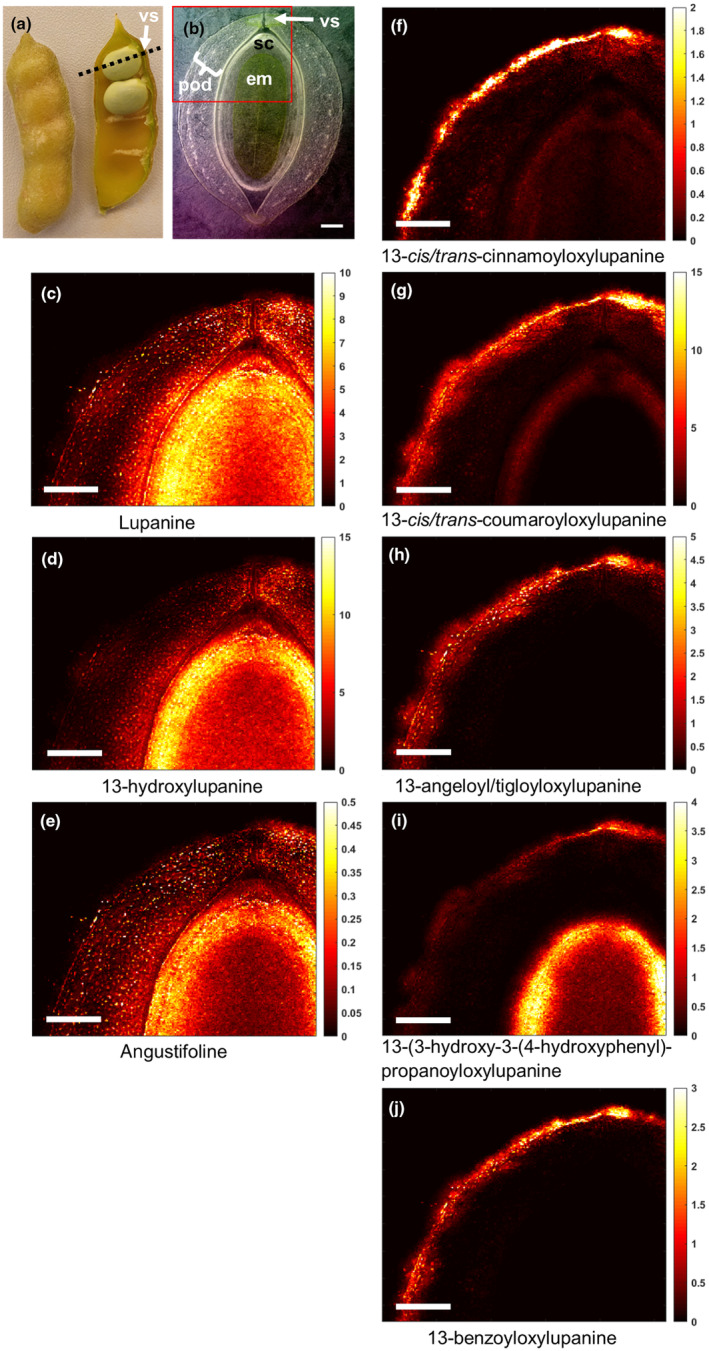
Transverse distribution of quinolizidine alkaloids (QAs) in a developing pod of narrow‐leafed lupin (*Lupinus angustifolius*), including an enclosed seed, as determined by matrix‐assisted laser desorption ionization mass spectrometry imaging (MALDI‐MSI). The pod was harvested at *c*. 30 DAA. (a) NLL pod with dotted line illustrating the analyzed transverse plane. (b) Bright‐field microscopy image with red box denoting the area that was further analyzed by high‐res MALDI‐MSI (bar, 1 mm). vs, ventral suture; pod, pod wall; sc, seed coat; em, seed embryo. (c–j) Individual MALDI‐MS images of QAs at 20 μm spatial resolution (bar, 1 mm). Each image was obtained by selecting the exact mass of the protonated QA (± 5 ppm, Supporting Information Table S1) and normalizing by the total ion current. The color scale represents signal intensity.

We imaged a section of a pod with seeds inside at 30 d after anthesis (DAA), which is *c*. halfway through seed development. At this point, the pods still contain relatively high levels of QAs, and these are expected to fall rapidly later in seed development (Otterbach *et al*., 2019). As the pod is bilaterally symmetrical, we imaged a representative portion containing the ventral suture (where the funiculus connects the pod to the seed) (Fig. 3a,b). The core QAs displayed an even distribution throughout the pod tissue (Fig. 3c–e), and again, the esterified QAs were predominantly in the epidermis (Fig. 3f–j). Interestingly, we observed a high amount of core QAs in the seed coat of the seed compared to the embryo (Fig. 3c–e,i). This could be indicative of an intermediate stage of long‐distance transport, in which QAs accumulate in the seed coat before being transferred to the embryo at near maturity. Finally, the esterified QA 13‐(3‐hydroxy‐3‐(4‐hydroxyphenyl)‐propanoyloxylupanine) was observed both in the pod epidermis as well as in the epidermis of the embryo.

### Tissue‐specific localization of biosynthetic gene expression

We used laser‐capture microdissection (LCM) to generate a tissue‐specific RNA‐Seq dataset from NLL leaves, stems, and developing pods. For each organ, we collected cells from as many tissues as possible, the limiting factors being the size of cells/tissues and their interconnectivity. This resulted in a different number of tissues collected for each organ, always including the epidermis (site of QA ester accumulation), the chloroplastic parenchyma (hypothesized site of QA biosynthesis), and the vasculature (involved in the long‐distance transport of QAs). After RNA extraction and Illumina sequencing, we quantified the expression of transcripts using the LCM‐RNA‐Seq data together with the coding sequences from a previously published NLL transcriptome (Yang *et al*., 2017), giving a final LCM‐RNA‐Seq gene expression matrix. We made this gene expression data available as an eFP browser (bar.utoronto.ca/efp_lupin) (Waese *et al*., 2017), where the expression of any NLL coding sequence can be easily visualized in transverse sections of leaves, stems, and developing pods. In the eFP browser, we also included the previously published RNA‐Seq dataset from eight whole NLL organs: leaf, stem, pedicel, flower, root, small pod including seeds, big pod, and big seed (Yang *et al*., 2017).

#### Leaf

**Fig. 4:**
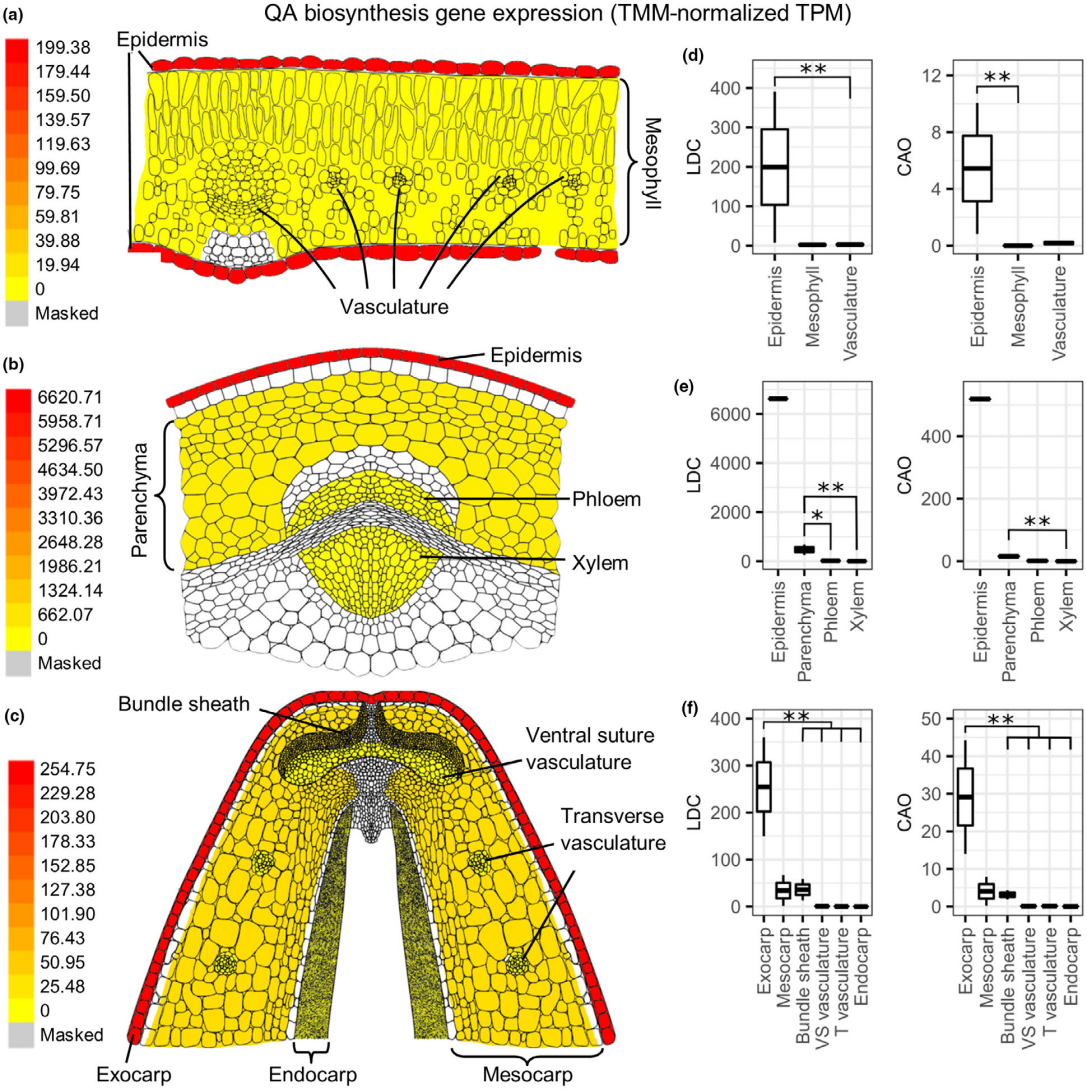
Expression of quinolizidine alkaloid (QA) biosynthetic genes *lysine decarboxylase* (*LDC*) and *copper amine oxidase* (*CAO*) in organs of narrow‐leafed lupin (*Lupinus angustifolius*) as determined by laser‐capture microdissection coupled to RNA‐Seq. (a–c) Heat maps of average expression of *LDC* (Luan_Oskar_PB12_103708) in leaf (a), stem (b), and developing pod (c) as shown on the Lupin eFP Browser. The scale on the left represents TMM‐normalized transcripts per million (TPM) values. The corresponding heat maps for *CAO* (Luan_Oskar_PB23_106386) are similar and are not depicted here. (d–f) Graphical representation of *LDC* and *CAO* expression in leaf (d), stem (e), and developing pod (f). Box plots represent the mean (line within box) as well as minimum and maximum values (whiskers) of two biological replicates (stem epidermis has one replicate). Asterisks indicate a significant difference as determined by differential gene expression analysis (DESeq2; Wald test): *, *P*_adj_ < 0.05; **, *P*_adj_ < 0.01.

Due to the small size of the cells/tissues in NLL leaves, it was only possible to separately collect three tissues: epidermis (abaxial and adaxial together), mesophyll, and vascular bundles. The latter included midvein and transverse vascular bundles, both of which encompassed phloem, xylem, and bundle sheath cells. The QA biosynthetic genes *LDC* and *CAO* were highly expressed in the leaf epidermis (Fig. 4a,d), and differential expression analysis revealed that *LDC* was significantly more highly expressed in epidermis compared to vasculature (log_2_ fold change = 6.18; *P*_adj_ = 0.006), and that *CAO* was significantly more highly expressed in epidermis vs mesophyll (log_2_ fold change = 10.44; *P*_adj_ < 0.001). The expression of *LDC* and *CAO* did not differ significantly between mesophyll and vasculature. As it is possible to peel abaxial (but not adaxial) epidermis from NLL leaves, we were able to isolate RNA from abaxial epidermis and verify the high expression of these QA biosynthetic genes by quantitative polymerase chain reaction. Indeed, *LDC* and *CAO* were significantly more highly expressed in the abaxial epidermis compared to the rest of the leaves (without abaxial epidermis), being 120 and 240 times more highly expressed, respectively (Fig. S6).

#### Stem

From stem tissue sections we collected epidermis, parenchyma, phloem, and xylem. While only the outer 1–3 cell layers of stem parenchyma have chloroplasts (Fig. S7), these cells were difficult to collect separately, and thus all the stem parenchyma was collected together. Additionally, it was difficult to determine the interface between phloem and vascular cambium cells, and therefore, our phloem samples likely contained a small proportion of vascular cambium. Notably, however, we were able to separate the phloem from the xylem for this organ.

Unfortunately, as one RNA sample for stem epidermis failed library preparation, it was not possible to carry out statistical comparisons involving the stem epidermis. Nevertheless, *LDC* and *CAO* did have very high expression in stem epidermis compared to parenchyma, phloem, and xylem (Fig. 4b,e). Additionally, *LDC* was significantly more highly expressed in parenchyma compared to phloem and xylem (phloem: log_2_ fold change = 4.82, *P*_adj_ = 0.015; xylem: log_2_ fold change = 6.99, *P*_adj_ < 0.001), and *CAO* was significantly more highly expressed in parenchyma compared to xylem (log_2_ fold change = 9.25, *P*_adj_ < 0.001).

#### Developing pod

Due to the large size and complexity of NLL pod cells/tissue, it was possible to collect a wider variety of tissues from the developing pod. We collected exocarp (outer epidermis), mesocarp (parenchyma without vasculature), bundle sheath, vasculature at the ventral suture, vasculature transversing the mesocarp, and endocarp (inner pod layer comprised of epidermis, thin‐walled parenchyma and sclerenchyma). *LDC* and *CAO* displayed high expression in the exocarp (outer epidermis) (Fig. 4c,f), with both genes being significantly more highly expressed in this tissue compared to all others (log_2_ fold change = 2.86–12.66, *P*_adj_ < 0.001) with the exception of the mesocarp.

### Visualization of chloroplasts in the epidermis of leaves, stems, and pods

**Fig. 5:**
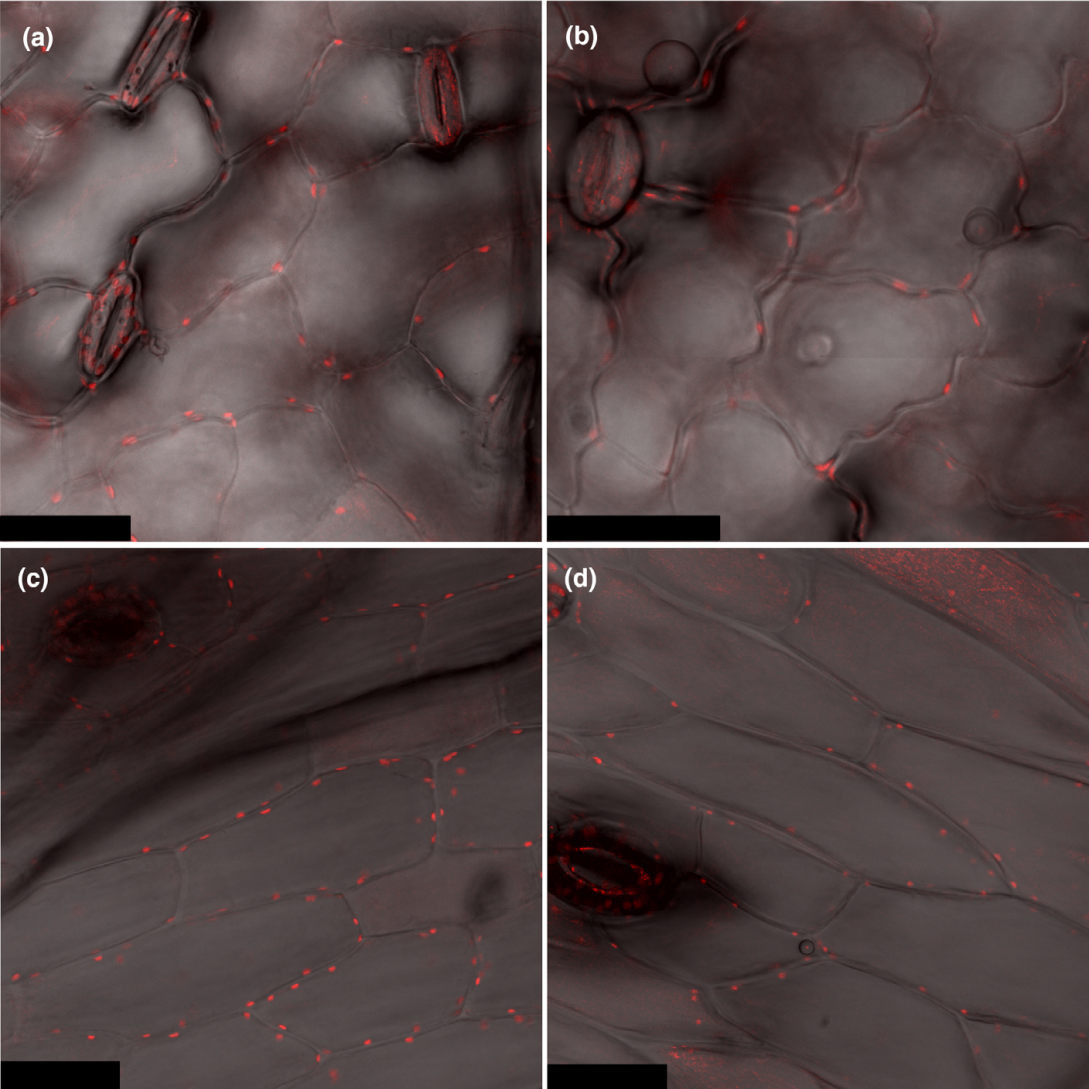
Visualization of chloroplasts in epidermal cells of narrow‐leafed lupin (*Lupinus angustifolius*). In each panel, Chl autofluorescence (red) is overlaid on the respective bright‐field microscopy image (grey). Black bars represent a distance of 40 μm. (a) Leaf abaxial epidermis. (b) Leaf adaxial epidermis. (c) Stem epidermis. (d) Developing pod.

Due to the unexpected result of *LDC* and *CAO* being highly expressed in the epidermis of leaves, stems, and pods, we probed these tissues for the presence of chloroplasts, where LDC has been found to localize intracellularly (Wink & Hartmann, 1982; Bunsupa *et al*., 2012). To do so we imaged Chl autofluorescence in the respective epidermal cells using confocal laser scanning microscopy. Indeed, the epidermis of all three organs contained chloroplasts (Fig. 5), although they were smaller and fewer in number than in the underlying cell layers (*z*‐stacks; Videos [Link], [Link]). In addition, some structures correlating to the areas of Chl autofluorescence in epidermal cells could be seen in the bright‐field microscopy images alone (Fig. S8).

### Probing the sites of QA biosynthesis in leaves via precursor feeding studies

Finally, to probe the sites of QA biosynthesis in leaves, we fed isotopically labeled L‐lysine (QA precursor) and analyzed labeled QAs via tissue dissection coupled to LC‐MS or via MALDI‐MSI. We had initially attempted to feed the labeled L‐lysine to isolated leaf epidermis, similarly to the method employed by Wink & Mende (1987), who fed radiolabeled cadaverine to isolated petiole epidermis. However, we found that even after a short incubation in media (6 h), the epidermis was nearly devoid of QAs, where it had previously had high levels of QAs before the incubation (Fig. S9). As an alternative approach, we fed the isotopically labeled L‐lysine to whole, detached leaves via the petiole and dissected the leaves for labeled QA analysis at various time points after continuous feeding. The predicted incorporation of labeled L‐lysine into the structure of lupanine is shown in Fig. S10. As previously mentioned, it is straightforward to strip the abaxial epidermis from NLL leaves, but not the adaxial epidermis. Therefore, we collected the abaxial epidermis, the remaining leaf tissue (without abaxial epidermis), and a sample of ‘mesophyll’ (tissue from leaves without epidermis, likely containing some vasculature). To collect this last type of tissue, leaves stripped of the abaxial epidermis were frozen, and the mesophyll was isolated by carefully scraping off tissue with a scalpel blade, avoiding the adaxial epidermis.

**Fig. 6:**
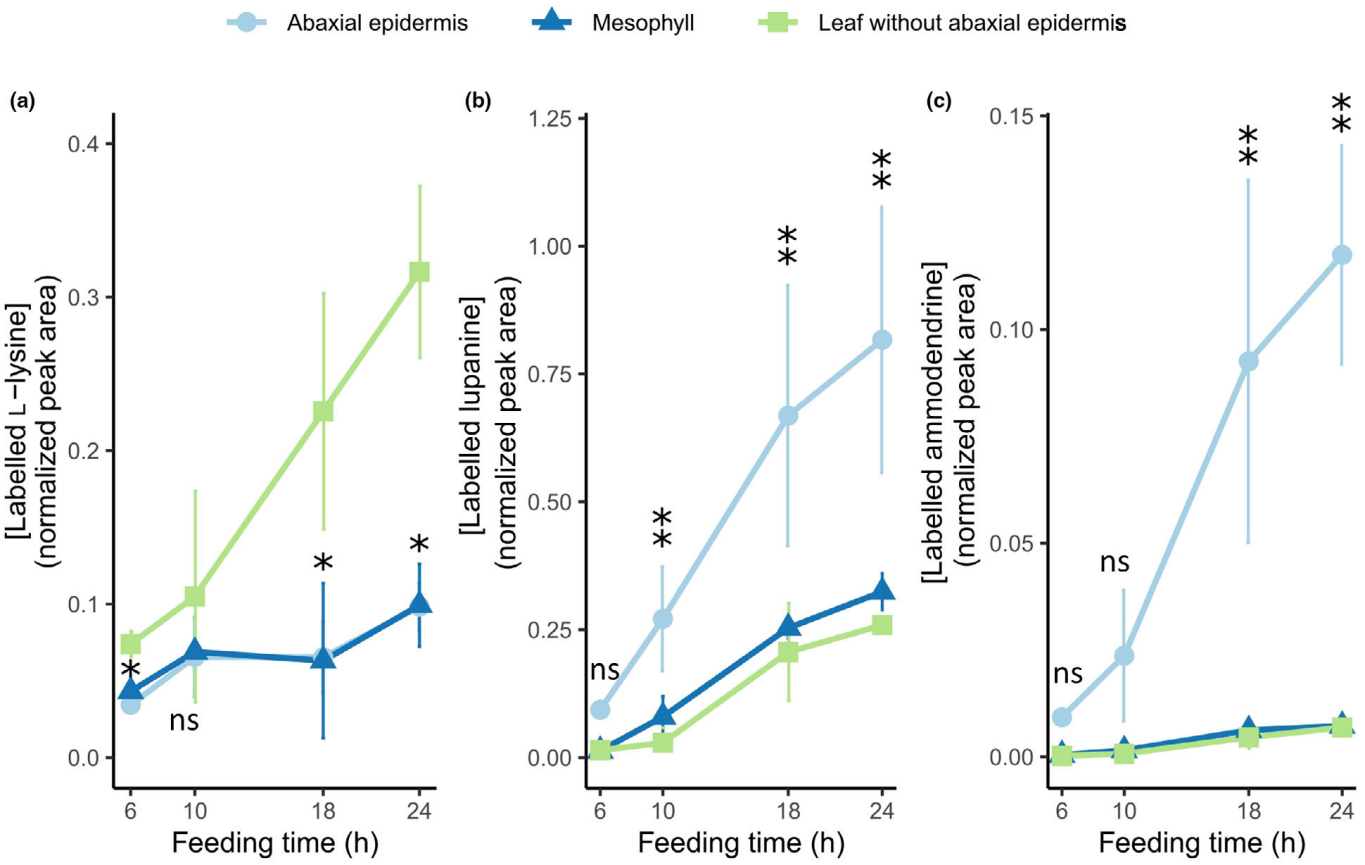
Accumulation of isotopically labeled compounds in tissue fractions of narrow‐leafed lupin (*Lupinus angustifolius*) leaves upon feeding with labeled L‐lysine. Whole leaves were fed via the cut petiole, and leaves were dissected at four time points after the start of the feeding period (6, 10, 18, and 24 h). The respective tissue fractions (abaxial epidermis, mesophyll, and leaf without abaxial epidermis) were analyzed by LC‐MS. Data points and error bars represent the mean and SD of 3 biological replicates. (a) Accumulation of the fed, labeled l‐lysine in the tissue fractions across time points. Single asterisks represent a significant difference between abaxial epidermis and the leaf without abaxial epidermis, and double asterisks represent significant differences between abaxial epidermis and both the leaf without abaxial epidermis and the mesophyll (*P*_adj_ < 0.05 on Tukey's test). ns, not significant. (b) Accumulation of the QA lupanine in the tissue fractions across time points. Significant differences are represented as described for (a). (c) Accumulation of ammodendrine (likely side product of the early QA pathway) in the tissue fractions across time points. Significant differences are represented as described for a, with the exception of the 10 h time point, for which a non‐parametric statistical test was used (*P*_adj_ < 0.05 on Dunn's test).

In these experiments, the fed, labeled L‐lysine was detected in all three tissue fractions, with the abaxial epidermis accumulating less than the remaining leaf tissue after 6, 18, and 24 h of feeding (Fig. 6a). By contrast, the abaxial epidermis accumulated more labeled lupanine than the remaining leaf tissue or the mesophyll, significantly so after 10, 18 and 24 h of feeding (Fig. 6b). In addition, the percentage of total lupanine that was labeled was significantly higher in the abaxial epidermis compared the remaining leaf tissue after 10, 18 and 24 h of feeding, and was also significantly higher compared to the mesophyll after 6 h of feeding (Fig. S11). Notably, after 24 h of feeding, nearly 50% of the lupanine in the abaxial epidermis was labeled (Fig. S11). Additionally, we could detect the presence of a likely side‐product of the early QA pathway, ammodendrine (Fig. S1; Mancinotti *et al*., 2022) in its labeled form (Fig. 6c). After 18 and 24 h of feeding, the levels of labeled ammodendrine were significantly higher in the abaxial epidermis than in the remaining leaf tissues and the mesophyll (Fig. 6c). We could also detect two other labeled core QAs (13‐hydroxylupanine and angustifoline) and a labeled QA ester (13‐*trans*‐cinnamoyloxylupanine) after 18 and 24 h of feeding (Fig. S12); however, levels were very low (barely detectable after 10 h of feeding). The levels of labeled 13‐hydroxylupanine and angustifoline did not differ significantly between the three tissue fractions, while the levels of labeled 13‐*trans*‐cinnamoyloxylupanine were significantly higher in the abaxial epidermis than the remaining leaf tissues and the mesophyll after 18 and 24 h of feeding (Fig. S12).

**Fig. 7:**
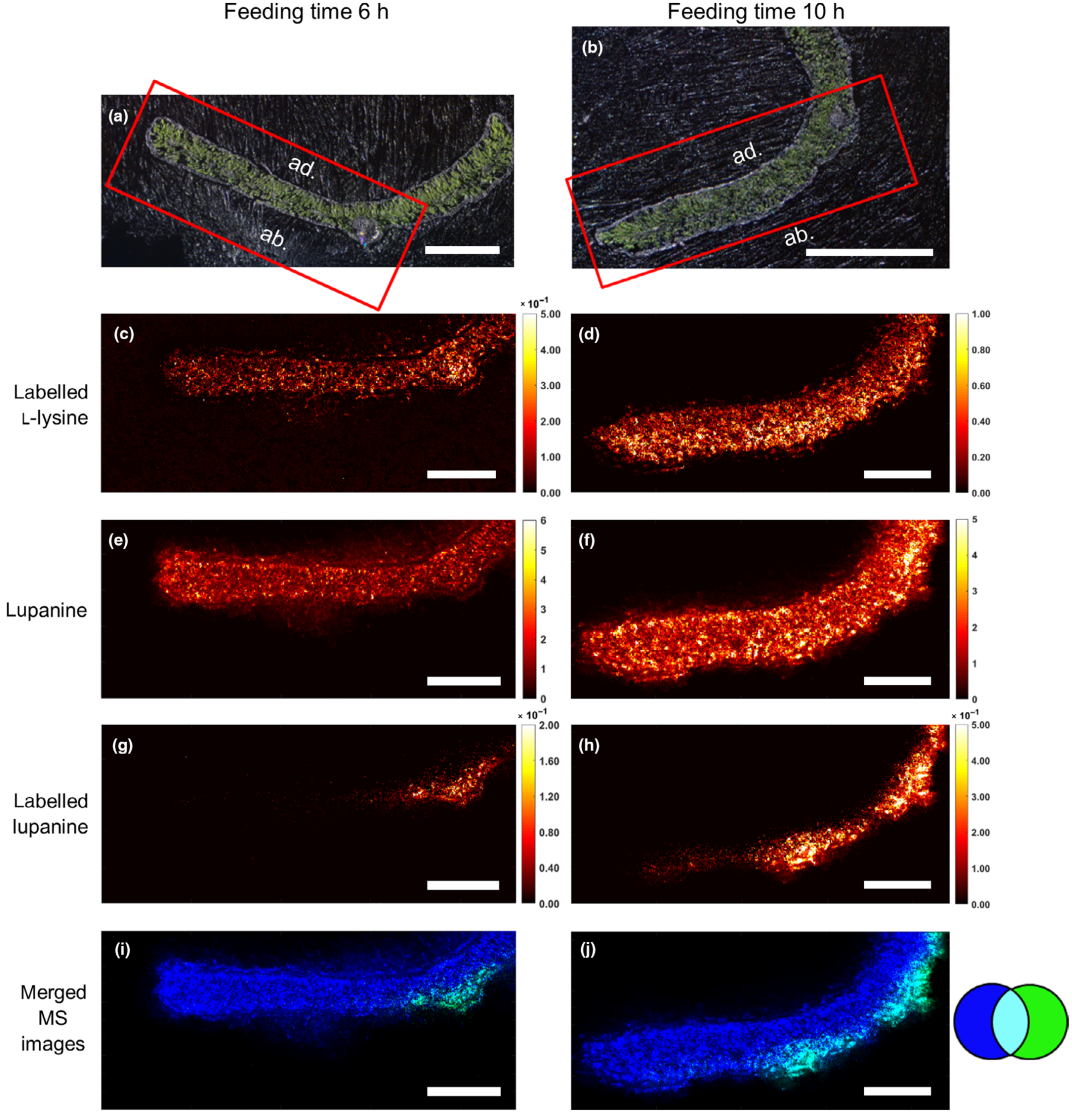
Distribution of isotopically labeled lupanine in transverse sections of narrow‐leafed lupin (*Lupinus angustifolius*) leaves at 6 h (left column) and 10 h (right column) after continuous feeding with isotopically labeled l‐lysine. Tissue sections were analyzed by high‐res matrix‐assisted laser desorption ionization mass spectrometry imaging (MALDI‐MSI) at 5 μm spatial resolution. Compounds were visualized by selecting the respective *m*/*z* ratios of their protonated forms (±5 ppm, Supporting Information Table S1) and normalizing by the total ion current. (a, b) Bright‐field microscopy images with red boxes representing the areas that were further analyzed (bar, 1 mm). (c, d) MALDI‐MSI visualization of labeled l‐lysine. The color scale (right) represents signal intensity (bar, 0.4 mm). (e, f) MALDI‐MSI visualization of unlabeled lupanine (same color scale; bar, 0.4 mm). (g, h) MALDI‐MSI visualization of labeled lupanine (same color scale; bar, 0.4 mm). (i, j) Merged and recolored MALDI‐MS images of unlabeled lupanine (blue) and labeled lupanine (green) (bar, 0.4 mm).

To generate further insights on the precise sites of biosynthesis, we generated MALDI‐MS images of transverse sections of the fed leaves for the early time points (6 and 10 h). While unlabeled lupanine and labeled L‐lysine were distributed throughout the leaf sections at both time points, the isotopically labeled lupanine showed a distinctive localization on the abaxial side of the leaf (Fig. 7). The accumulation began on the abaxial side around the midrib after 6 h of feeding and appeared to extend both laterally along the abaxial side and also toward the mesophyll after 10 h of feeding (Fig. 7). Images of a control membrane component (phosphatidylcholine 34 : 2) are shown in Fig. S13. A replicate of this MALDI‐MSI dataset is presented in Fig. S14. Negative controls were also imaged (leaves fed with unlabeled L‐lysine), and they gave no signal for both the labeled L‐lysine and the labeled lupanine as expected (Fig. S15). We also obtained one set of images of the leaves fed with labeled L‐lysine and harvested at the two later timepoints (18 and 24 h after the commencement of feeding) using a modified MALDI‐MSI protocol. These images also show the expansion of the signal for labeled lupanine from the abaxial side near the midrib towards the rest of the leaf (Fig. S16).

## Discussion

Here we show that QAs in NLL are made primarily in the epidermis of QA‐synthesizing organs (leaves, stems, and developing pods). Supporting evidence includes the higher expression of biosynthetic genes in the epidermis compared to other tissues (Fig. 4, S6) as well as the larger incorporation of label from a QA precursor into lupanine in the leaf epidermis compared to the rest of the leaf (Figs 6, 7, S11). For both lines of evidence, we collected results from two different experimental approaches. In the case of gene expression, the experimental approaches comprised LCM‐RNA‐Seq (Fig. 4) as well as manual leaf tissue dissection followed by quantitative polymerase chain reaction (Supporting Information Fig. S6). In the case of the precursor feeding studies, we analyzed the fed leaves via manual dissection coupled to LC‐MS (Figs 6, S11) as well as via MALDI‐MSI (Fig. 7).

Our findings stand in contrast to those of Wink & Mende (1987) who concluded that QAs were made in mesophyll tissue. In their study, the radiolabeled QA intermediate cadaverine was separately fed to intact petioles of *L. polyphyllus*, the stripped petiole epidermis, and the remaining tissue (‘mesophyll’). Radiolabeled lupanine was detected in intact petioles and in the ‘mesophyll’, but not in the epidermis. However, the authors did not report the levels of unlabeled QAs in this experiment. In our experience, when we attempted to feed isotopically labeled L‐lysine to stripped leaf epidermis, the high levels of unlabeled QAs originally present became almost undetectable in the course of 6 h (Fig. S9). It is possible that a similar loss of QAs occurred in the experiment by Wink and Mende, thus preventing the detection of radiolabeled QAs in the stripped epidermis (e.g. due to loss of cell viability, rapid QA metabolism, or efficient QA export). However, it should also be noted that NLL and *L. polyphyllus* derive from different geological lineages (Old World vs New World lupins), and have different life cycles (annual vs perennial), so there remains the possibility that differences in the localization of QA biosynthesis exist between the two distantly related species (Aïnouche *et al*., 2004). Further experiments are needed to determine whether the robust biosynthetic capacity that we observed in the NLL epidermis can be extrapolated to other *Lupinus* species, particularly for other agriculturally important Old World lupins such as *L. albus* and *L. luteus*.

Wink & Mende (1987) argued that epidermal cells are not able to make substantial amounts of QAs because they are usually devoid of chloroplasts – where the biosynthetic enzyme LDC localizes. Notably, in our study, we were able to observe chloroplasts in the epidermis of all biosynthetic organs of NLL (Fig. 5). While epidermal chloroplasts are often overlooked in higher plants, the presence of epidermal chloroplasts is particularly well documented for *Arabidopsis thaliana* and tobacco (Dupree *et al*., 1991; Barton *et al*., 2016). Already in 1879, it had been found that 85–95% of the 102 studied dicotyledonous species had epidermal chloroplasts, in particular, in the abaxial leaf epidermis (Stöhr, 1879). Although the function of epidermal chloroplasts is still not well understood, their size and number is smaller than those of mesophyll cells. In addition, their low Chl content has led to the assumption that their photosynthetic contribution is comparatively low (Barton *et al*., 2016). If the activity of LDC does indeed need chloroplast localization, the required chloroplasts do exist in the epidermis of NLL biosynthetic organs. It might be of interest, however, to investigate if LDC also localizes elsewhere in epidermal cells, in particular, in other plastid types. Interestingly, functional LDC can be expressed in hairy roots of *Nicotiana tabacum*, where it likely localized to the root's leucoplasts (Bunsupa *et al*., 2014). The high‐biosynthetic capacity of the NLL epidermis may arise from localization in different types of NLL epidermal plastids or even elsewhere in epidermal cells. In any case, it is worth noting that even though LDC was initially found to localize in chloroplasts purified from whole leaf fractions of *L. polyphyllus*, the activity of LDC was 2–3 orders of magnitude lower than that of the final enzyme of lysine biosynthesis (also localized to chloroplasts) (Wink & Hartmann, 1982). This is consistent with only a fraction of the purified chloroplasts – likely the epidermal fraction – being biosynthetic with respect to QAs.

Interestingly, our precursor feeding studies coupled to MALDI‐MSI suggest that QAs in NLL are synthesized in the abaxial and not the adaxial leaf epidermis (Fig. 7). This is supported by the quantitative polymerase chain reaction results from manually dissected leaves, which show that very little biosynthetic gene expression remains in the leaf after peeling the adaxial epidermis off (Fig. S6). It should be noted that, in our LCM‐RNA‐Seq experiment, we collected the abaxial and the adaxial leaf epidermis together. Thus, the depiction of *LDC* expression in both epidermal layers in Fig. 4(a) is due to our inability to discriminate between these layers in this experiment (rather than a representation of our running hypothesis). It is not clear why QA biosynthesis should take place mostly or only in the abaxial leaf epidermis. Leaf development is subject to strict regulation that establishes and maintains the abaxial–adaxial axis (Manuela & Xu, 2020). Since very little is known about the regulation of QA biosynthesis, further studies are needed to uncover any connections between abaxial–adaxial axis establishment and QA biosynthesis.

Our new finding that QAs are made primarily in the epidermis of QA‐synthesizing organs has important implications for the elucidation for the entire QA pathway, for which only the first few genes are known (Mancinotti *et al*., 2022). The LCM‐RNA‐Seq dataset generated in this study is a valuable resource for the discovery of QA biosynthesis genes, which are likely co‐expressed with *LDC* and *CAO* in the epidermis, at least until the formation of lupanine. The dataset can be used to identify candidate genes based on their expression, with greater power and resolution than previous RNA‐Seq datasets have allowed (such as that described in Yang *et al*., 2017). Co‐expression analyses have been instrumental in the identification of genes involved in plant specialized metabolism, as these are often under strict transcriptional regulation (Wisecaver *et al*., 2017).

**Fig. 8:**
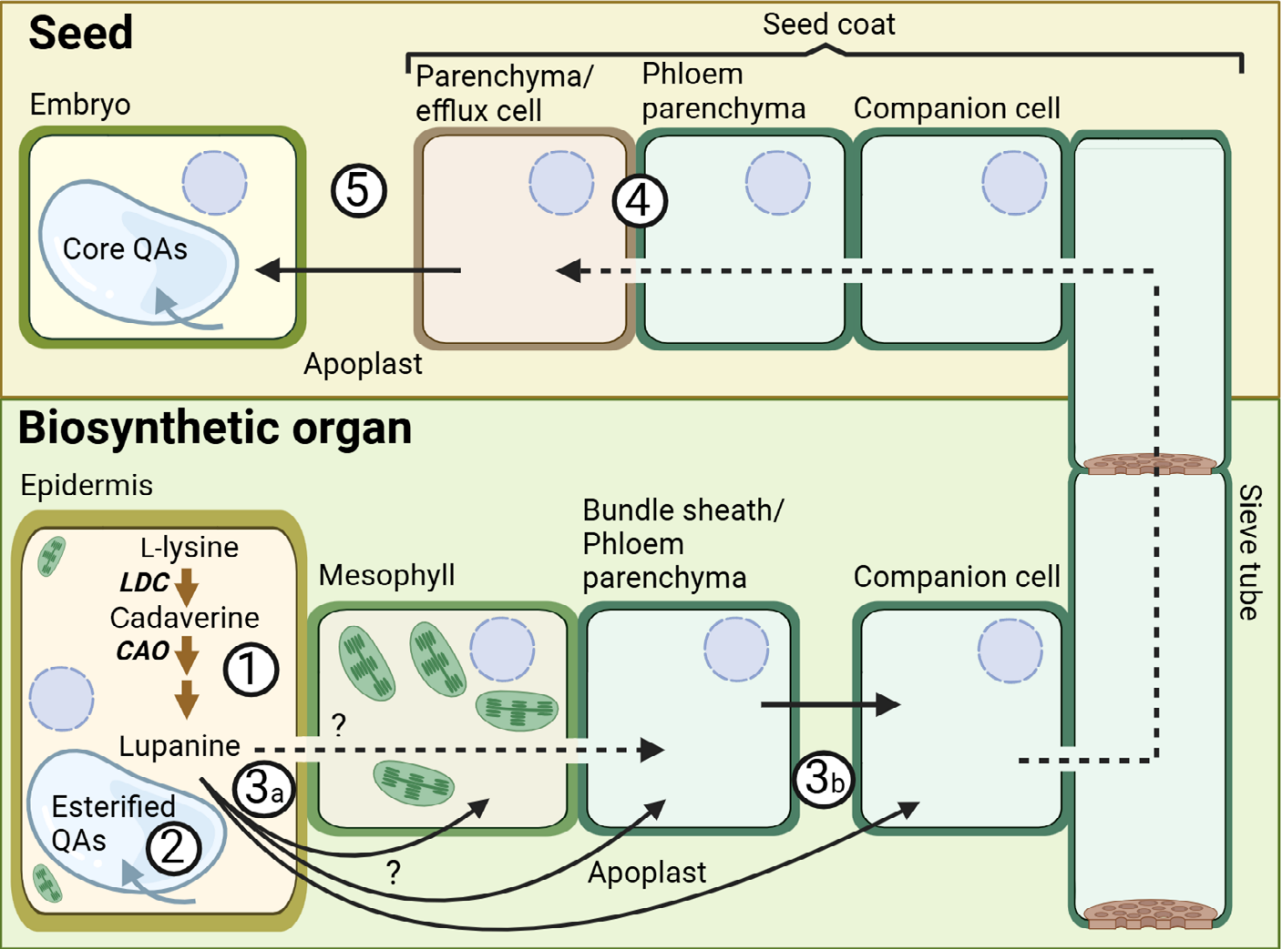
Spatial model for quinolizidine alkaloid (QA) biosynthesis and transport in narrow‐leafed lupin (NLL, *Lupinus angustifolius*). The aerial epidermis is a major site of QA biosynthesis, at least until the formation of lupanine (1) (pathway sub‐cellular localization is yet to be defined). The esterified QAs accumulate in the epidermis, likely within the vacuole (2) (Mende & Wink, 1987). The core QAs (e.g. lupanine) must move from the epidermis to the phloem‐associated cells for long‐distance transport to the seeds. It is yet to be shown whether this is via a symplastic or apoplastic route, or both (3a). The apoplastic phloem loading mechanism depicted (3b) is generally found in Fabaceae species (although not yet confirmed for NLL) (Bourquin *et al*., 1990; Wimmers & Turgeon, 1991). After long‐distance transport through the phloem, the QAs likely enter the seed coat cells through symplastic connections with the vasculature (4) (van Dongen *et al*., 2003; Tegeder, 2014). Finally, an apoplastic transport step is required for the QAs to accumulate in embryo cells (5) (Tegeder, 2014). Dotted black arrows represent symplastic transport, solid black arrows represent apoplastic transport, blue arrows represent transport into the vacuole, and brown arrows represent QA biosynthesis steps. This figure was created in BioRender (BioRender.com/a33n741).

While our findings show that lupanine is mostly made in the epidermis, it is unclear where subsequent steps of the QA biosynthetic pathway take place. It should, however, be noted that lupanine is the most abundant QA in both leaves and seeds of NLL (Wink *et al*., 1995), and it is the precursor of most, if not all, other QAs in NLL (Mancinotti *et al*., 2021). In our feeding experiments, we observed much higher signals for labeled lupanine than for any other labeled QA (Figs. 6, S12). However, our feeding experiments lasted at most 24 h, and thus, it is possible that longer feeding experiments would lead to a higher conversion towards 13‐hydroxylupanine and its esters. Notably, MALDI‐MSI revealed that QA esters localize primarily to the epidermis of QA‐synthesizing organs (Figs 1, 2, 3), suggesting that QA esters are synthesized in the epidermis of these organs and remain there. In accordance, we observed trace formation of labeled 13‐*trans‐*cinnamoyloxylupanine in the epidermis (Fig. S12). Further experiments should be carried out to test this hypothesis (e.g. longer feeding experiments). By contrast, the core QAs seem to be transported to the underlying cell layers, as observed for labeled lupanine in the MALDI‐MS images of precursor‐fed leaves (Fig. 7). Eventually, the core QAs must be transported into the phloem (Lee *et al*., 2006) for their translocation to the seeds (Otterbach *et al*., 2019). We present our new working hypothesis in Fig. 8. The molecular mechanisms by which this transport pathway takes place remain to be discovered. A full understanding of QA biosynthesis and transport in NLL may allow for the targeted removal of QAs from the seeds to increase the nutritional value of the high‐protein seeds.

While the core QAs accumulate evenly across tissues and are transported long‐distance to the seeds, the esterified QAs are found primarily in the epidermis of biosynthetic organs (Figs 1, 2, 3). This suggests that the esterified QAs play a distinct biological role in the aboveground epidermis. QAs have long been thought to play a role in plant protection, particularly against insect pests. Interestingly, the QA ester 13‐angeloyl/tigloyloxylupanine has been reported to be a more effective antimicrobial agent than the core QA lupanine (Wink, 1984). In addition, both 13‐angeloyl/tigloyloxylupanine and 13‐*cis*/*trans*‐coumaroyloxylupanine were found to be highly deterrent against spruce budworm, whereas the core QAs were not (Bentley *et al*., 1984). The study of the molecular mechanisms for biosynthesis and storage of QA esters in the NLL epidermis will help elucidate their distinct physiological roles.

### Conclusion

Here we show for the first time that the QAs in NLL are mainly synthesized in the epidermis of biosynthetic organs (leaves, stems, and developing pods). While the core QAs are transported to other tissues and ultimately translocated to the seeds, the esterified QAs accumulate to high levels in the epidermis of biosynthetic organs. Furthermore, we provide a valuable tissue‐specific LCM‐RNA‐Seq dataset, which we have made available as a Lupin eFP Browser together with a previously generated organ‐specific RNA‐Seq dataset. Our work uncovers new aspects of QA biosynthesis and transport, setting the stage for the discovery of the underlying molecular mechanisms.

## Competing interests

None declared.

## Author contributions

KMF and FG‐F conceived the research plan. KMF, MDBBL and NM carried out the experiments, analyzed the data and prepared the figures. NJP, EE and AP generated the Lupin eFP Browser. AS, HHN‐E, NB and CJ provided supervision and contributed with data interpretation. FG‐F coordinated and supervised the project. KMF wrote the manuscript with input from all authors.

## Disclaimer

The New Phytologist Foundation remains neutral with regard to jurisdictional claims in maps and in any institutional affiliations.

## Supporting information


**Fig. S1** The quinolizidine alkaloid biosynthetic pathway in narrow‐leafed lupin (*Lupinus angustifolius*).
**Fig. S2** RNA integrity traces of RNA isolated from laser‐capture microdissected cells/tissues of narrow‐leafed lupin (*Lupinus angustifolius*) as measured on an Agilent 2100 Bioanalyzer using the Agilent RNA 6000 Pico Kit.
**Fig. S3** Transverse distribution of phosphatidylcholine 34 : 2 [PC(34 : 2)] in biosynthetic tissues of narrow‐leafed lupin (*Lupinus angustifolius*) as determined by matrix‐assisted laser desorption ionization mass spectrometry imaging at a spatial resolution of 5 μm.
**Fig. S4** Mass spectra of selected pixels within the narrow‐leafed lupin (*Lupinus angustifolius*) MALDI‐MSI dataset.
**Fig. S5** Example of the replication of the results presented in Fig. 1.
**Fig. S6** Expression of two quinolizidine alkaloid biosynthetic genes in the leaf abaxial epidermis of narrow‐leafed lupin (*Lupinus angustifolius*) compared to the rest of the leaf without abaxial epidermis as determined by quantitative polymerase chain reaction.
**Fig. S7** Fluorescence microscopy image of a cross section of a stem of narrow‐leafed lupin (*Lupinus angustifolius*).
**Fig. S8** Bright‐field microscopy images of intact biosynthetic organs of narrow‐leafed lupin (*Lupinus angustifolius*).
**Fig. S9** Normalized relative peak area of unlabeled quinolizidine alkaloids in leaf abaxial epidermis of narrow‐leafed lupin (*Lupinus angustifolius*) after 0 h and 6 h incubation in media (B5 medium, 2% sucrose and 1–5 mM labeled lysine) as determined by LC‐MS.
**Fig. S10** Incorporation of the isotopically labeled L‐lysine used in the feeding experiments into the tetracyclic quinolizidine alkaloid backbone (derived from three units of L‐lysine).
**Fig. S11** Percentage of total amount of lupanine with isotopic label in tissue fractions of narrow‐leafed lupin (*Lupinus angustifolius*) leaves upon feeding with labeled L‐lysine.
**Fig. S12** Trace accumulation of three isotopically labeled quinolizidine alkaloids in tissue fractions of narrow‐leafed lupin (*Lupinus angustifolius*) leaves upon feeding with labeled L‐lysine.
**Fig. S13** Distribution of control membrane component phosphatidylcholine 34 : 2 [PC(34 : 2)] in narrow‐leafed lupin (*Lupinus angustifolius*) transverse leaf sections at 6 and 10 h after feeding with isotopically labeled L‐lysine.
**Fig. S14** Replicate of the experiment shown in Fig. 7 (MALDI‐MS imaging of leaves fed with isotopically labeled L‐lysine at the two first time points).
**Fig. S15** Distribution of phosphatidylcholine 34 : 2 [PC(34 : 2), control membrane component] and labeled versions of L‐lysine and lupanine (negative controls) in transverse sections of narrow‐leafed lupin (*Lupinus angustifolius*) leaves after feeding with unlabeled L‐lysine.
**Fig. S16** Distribution of isotopically labeled L‐lysine and isotopically labeled lupanine in transverse leaf sections of narrow‐leafed lupin (*Lupinus angustifolius*) at 18 h (images on the left) and 24 h (images on the right) after feeding with isotopically labeled L‐lysine.
**Table S1** List of compounds examined in *Lupinus angustifolius* (NLL) tissues via MALDI‐MSI and/or LC‐MS.

**Video S1** Chlorophyll autofluorescence (red) in the narrow‐leafed lupin (*Lupinus angustifolius*) leaf abaxial epidermis and underlying cell layers (z‐stack).

**Video S2** Chlorophyll autofluorescence (red) in the narrow‐leafed lupin (*Lupinus angustifolius*) leaf adaxial epidermis and underlying cell layers (z‐stack).

**Video S3** Chlorophyll autofluorescence (red) in the narrow‐leafed lupin (*Lupinus angustifolius*) stem epidermis and underlying cell layers (z‐stack).

**Video S4** Chlorophyll autofluorescence (red) in the narrow‐leafed lupin (*Lupinus angustifolius*) developing pod epidermis and underlying cell layers (z‐stack).
Please note: Wiley is not responsible for the content or functionality of any Supporting Information supplied by the authors. Any queries (other than missing material) should be directed to the *New Phytologist* Central Office.

## Data Availability

The MSI data generated during this study are available on METASPACE (https://metaspace2020.eu/project/lupinus_angustifolius_Frick‐et‐al). The RNA‐Seq data here generated are available on the NCBI Sequence Read Archive (SRA) (BioProject PRJNA943121). The Lupin eFP Browser is available at https://bar.utoronto.ca/efp_lupin/cgi‐bin/efpWeb.cgi.
