# Supplementary material for: The aerial epidermis is a major site of quinolizidine alkaloid biosynthesis in narrow‐leafed lupin
Source: New Phytol. 2025 Jan 14;245(5):2052–68. doi: 10.1111/nph.20384 (PMC11798894; doi:10.1111/nph.20384)
Supplement: Supplementary file 1 — Fig. S1 The quinolizidine alkaloid biosynthetic pathway in narrow‐leafed lupin (Lupinus angustifolius). Fig. S2 RNA integrity traces of RNA isolated from laser‐capture microdissected cells/tissues of narrow‐leafed lupin (Lupinus angustifolius) as measured on an Agilent 2100 Bioanalyzer using the Agilent RNA 6000 Pico Kit. Fig. S3 Transverse distribution of phosphatidylcholine 34 : 2 [PC(34 : 2)] in biosynthetic tissues of narrow‐leafed lupin (Lupinus angustifolius) as determined by matrix‐assisted laser desorption ionization mass spectrometry imaging at a spatial resolution of 5 μm. Fig. S4 Mass spectra of selected pixels within the narrow‐leafed lupin (Lupinus angustifolius) MALDI‐MSI dataset. Fig. S5 Example of the replication of the results presented in Fig. 1. Fig. S6 Expression of two quinolizidine alkaloid biosynthetic genes in the leaf abaxial epidermis of narrow‐leafed lupin (Lupinus angustifolius) compared to the rest of the leaf without abaxial epidermis as determined by quantitative polymerase chain reaction. Fig. S7 Fluorescence microscopy image of a cross section of a stem of narrow‐leafed lupin (Lupinus angustifolius). Fig. S8 Bright‐field microscopy images of intact biosynthetic organs of narrow‐leafed lupin (Lupinus angustifolius). Fig. S9 Normalized relative peak area of unlabeled quinolizidine alkaloids in leaf abaxial epidermis of narrow‐leafed lupin (Lupinus angustifolius) after 0 h and 6 h incubation in media (B5 medium, 2% sucrose and 1–5 mM labeled lysine) as determined by LC‐MS. Fig. S10 Incorporation of the isotopically labeled L‐lysine used in the feeding experiments into the tetracyclic quinolizidine alkaloid backbone (derived from three units of L‐lysine). Fig. S11 Percentage of total amount of lupanine with isotopic label in tissue fractions of narrow‐leafed lupin (Lupinus angustifolius) leaves upon feeding with labeled L‐lysine. Fig. S12 Trace accumulation of three isotopically labeled quinolizidine alkaloids in tissue fractions of narr [file NPH-245-2052-s001.pdf]

New Phytologist

Supporting Information

The aerial epidermis is a major site of quinolizidine alkaloid biosynthesis in narrow-leafed lupin

Karen Michiko Frick[1], Marcus Daniel Brandbjerg Bohn Lorensen[1,2], Nikola Micic[1], Eddi Esteban[3], Asher Pasha[3], Alexander Schulz[4], Nicholas James Provart[3], Hussam Hassan Nour-Eldin[5], Nanna Bjarnholt[1], Christian Janfelt[2], and Fernando Geu-Flores[1]*.

*Correspondence: feg@plen.ku.dk

[1]Section for Plant Biochemistry and Copenhagen Plant Science Centre, Department of Plant and Environmental Sciences, University of Copenhagen, Frederiksberg, Denmark

[2]Department of Pharmacy, University of Copenhagen, Copenhagen, Denmark

[3]Department of Cell and Systems Biology/Centre for the Analysis of Genome Evolution and Function, University of Toronto, Toronto, Ontario, Canada

[4]Section for Transport Biology, Department of Plant and Environmental Sciences, University of Copenhagen, Frederiksberg, Denmark

[5]Section for Plant Molecular Biology, Department of Plant and Environmental Sciences, University of Copenhagen, Frederiksberg, Denmark

Article accepted on 07 December 2024

Contents:

- Table S1
- Figures S1-S16
- Legends of videos S1-S4

Table S1. List of compounds examined in *Lupinus angustifolius* (NLL) tissues via MALDI-MSI and/or LC-MS. The associated formulas, structures, and mass-to-charge ratios (*m/z*) used for analysis are shown.

Compound	Chemical formula (analyzed form)	Chemical structures of QAs, precursors or intermediates	*m/z*
PC(34:2)	$[PC(34:2)+K]^+$		796.5253
lupanine	$C_{15}H_{25}N_2O^+$ $[M+H]^+$	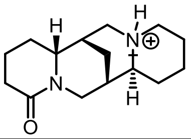	249.1961
Isotopically labelled lupanine	$^{13}C_{15}H_{25}{}^{15}N_2O^+$ $[M+H]^+$	As above	266.2405
13-hydroxylupanine	$C_{15}H_{25}N_2O_2^+$ $[M+H]^+$	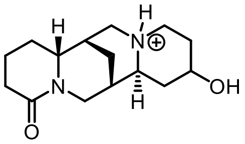	265.1911
Isotopically labelled 13-hydroxylupanine	$^{13}C_{15}H_{25}{}^{15}N_2O_2^+$ $[M+H]^+$	As above	282.2354
angustifoline	$C_{14}H_{23}N_2O^+$ $[M+H]^+$	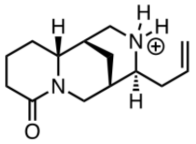	235.1805
Isotopically labelled angustifoline	$^{13}C_{14}H_{23}{}^{15}N_2O^+$ $[M+H]^+$	As above	251.2215
13-angeloyl/ tigloyloxylupanine*	$C_{20}H_{31}N_2O_3^+$ $[M+H]^+$	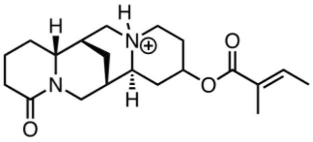	347.2329
13-*cis*-cinnamoyloxylupanine‡	$C_{24}H_{31}N_2O_3^+$ $[M+H]^+$	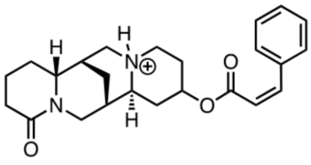	395.2329
13-*trans* -cinnamoyloxylupanine‡	$C_{24}H_{31}N_2O_3^+$ $[M+H]^+$	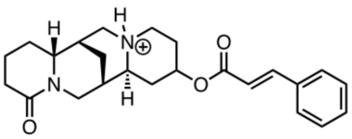	395.2329
Isotopically labelled 13-*trans* -cinnamoyloxylupanine	$^{13}C_{15}C_9H_{31}{}^{15}N_2O_3^+$ $[M+H]^+$	As above	412.2773

13-*cis/trans*-coumaroyloxylupanine*	$C_{24}H_{31}N_2O_4^+$ [M+H]$^+$	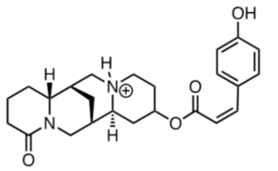	411.2278
13-(3-hydroxy-3-(4-hydroxyphenyl)-propanoyloxylupanine	$C_{24}H_{33}N_2O_5^+$ [M+H]$^+$	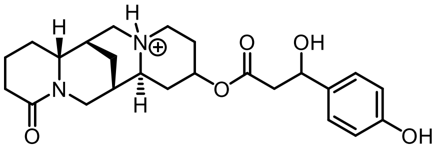	429.2384
13-benzoyloxylupanine	$C_{22}H_{29}N_2O_3^+$ [M+H]$^+$	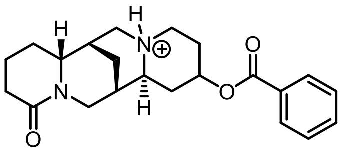	369.2173
ammodendrine	$C_{12}H_{21}N_2O^+$ [M+H]$^+$	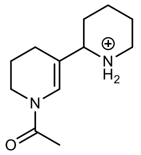	209.1648
Isotopically labelled ammodendrine	$^{13}C_{10}C_2H_{21}{}^{15}N_2O^+$ [M+H]$^+$	As above	221.1925
L-lysine (examined via MALDI-MSI)	$C_6H_{15}N_2O_2^+$ [M+H]$^+$	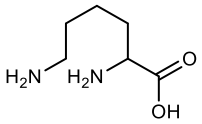	147.1128
Isotopically labelled L-lysine (examined via MALDI-MSI)	$^{13}C_6H_{15}{}^{15}N_2O_2^+$ [M+H]$^+$	As above	155.1270
L-lysine (examined via LC-MS)	$C_6H_{12}NO_2^+$ $[M – NH_3]^+$	Structure undetermined	130.0863
Isotopically labelled L-lysine (examined via LC-MS)	$^{13}C_6H_{12}{}^{15}NO_2^+$ $[M – NH_3]^+$	As above	137.1034

*Only one of these isomer pairs accumulates in NLL, and we did not determine which one.

‡These isomers could be differentiated via LC-MS as described in Otterbach *et al.* (2019).

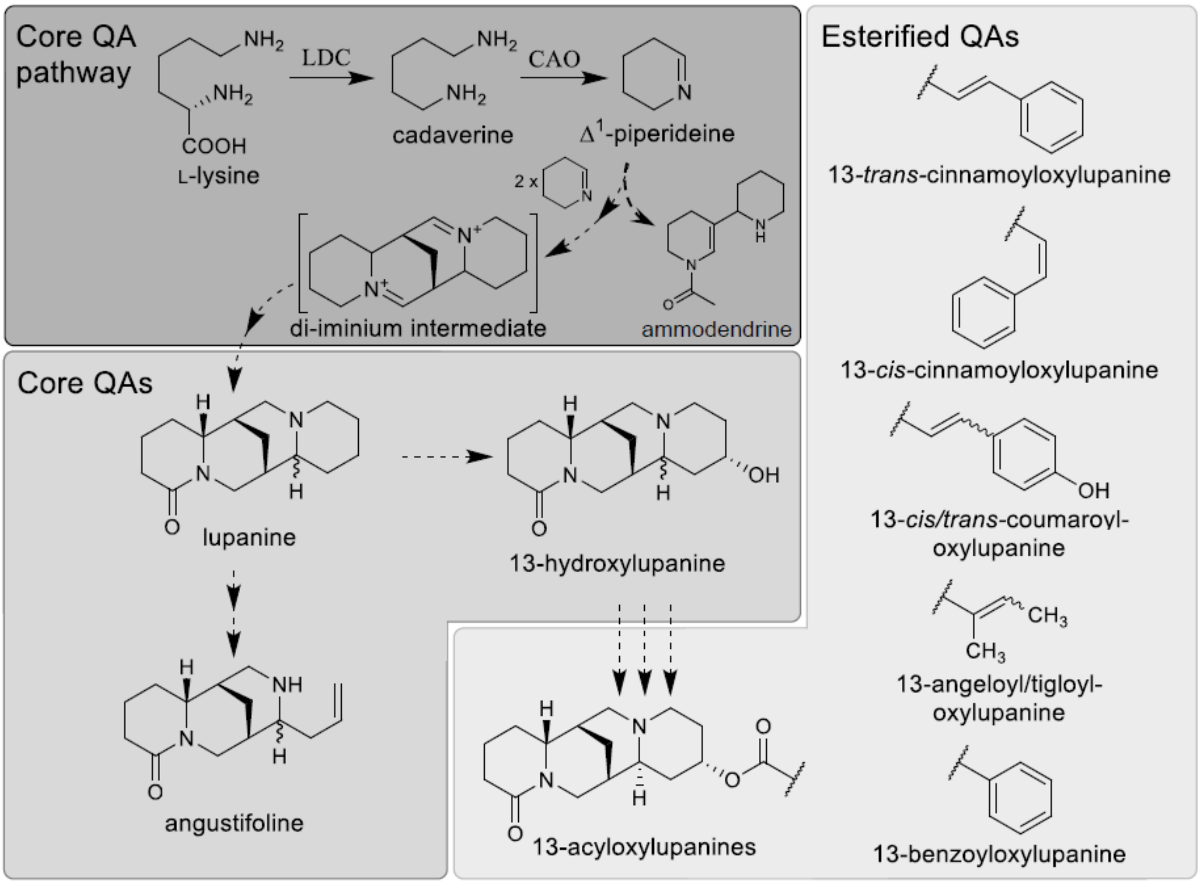

Fig. S1. The quinolizidine alkaloid (QA) biosynthetic pathway in narrow-leafed lupin (*L. angustifolius*). QAs are synthesized from L-lysine, which is decarboxylated by lysine decarboxylase (LDC) to yield cadaverine. Cadaverine is then oxidatively deaminated to Δ^1-piperideine by copper amine oxidase (CAO). Three Δ^1-piperideine molecules are then joined to form a four-ring, putative di-iminium intermediate. Ammodendrine is likely an early by-product of QA biosynthesis. Lupanine is the simplest QA that accumulates in NLL, and is the precursor for most, if not all, other QAs in NLL. Lupanine, 13-hydroxylupanine, and angustifoline constitute the main 'core' QAs in NLL. 13-hydroxylupanine can be further modified to form an array of 'esterified' QAs. Plain and dashed arrows indicate, respectively, characterized and predicted steps. This figure has been modified from Mancinotti *et al.,* (2021), and is presented with permission from the authors.

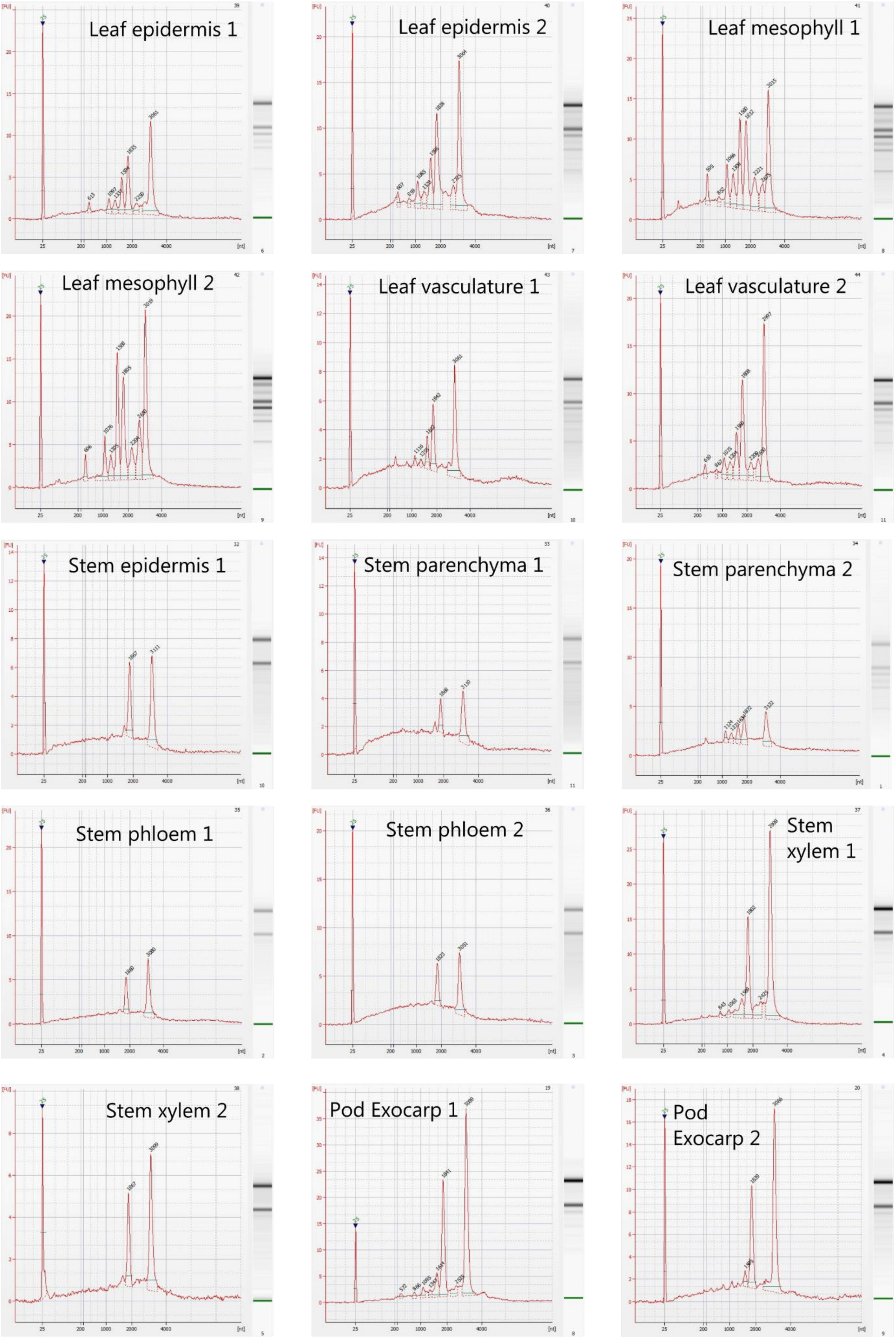
Leaf epidermis 1
Leaf epidermis 2
Leaf mesophyll 1
Leaf mesophyll 2
Leaf vasculature 1
Leaf vasculature 2
Stem epidermis 1
Stem parenchyma 1
Stem parenchyma 2
Stem phloem 1
Stem phloem 2
Stem xylem 1
Stem xylem 2
Pod Exocarp 1
Pod Exocarp 2

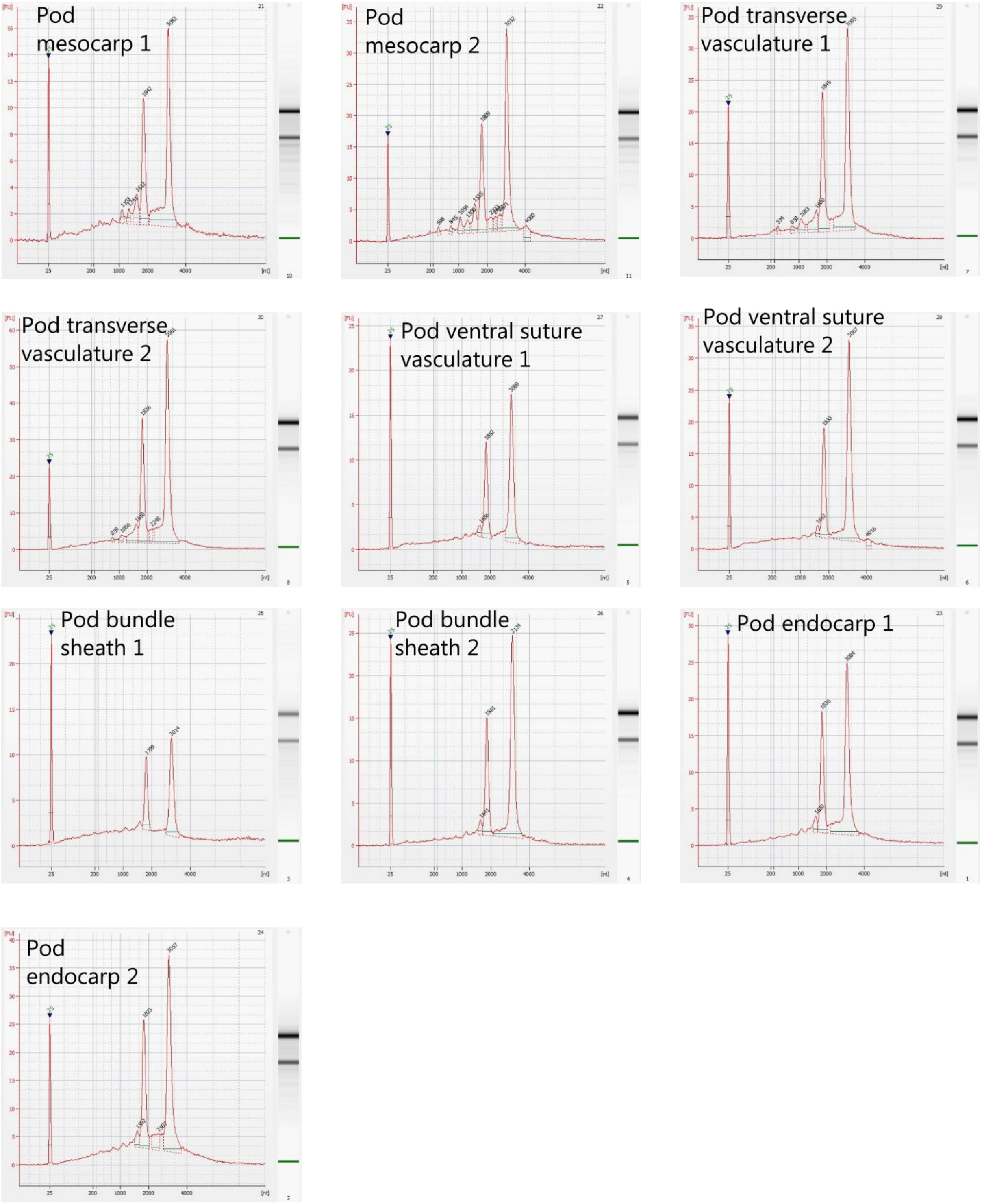

Fig. S2. RNA integrity traces of RNA isolated from laser-capture microdissected cells/tissues of narrow-leafed lupin (*L. angustifolius*) as measured on an Agilent 2100 Bioanalyzer using the Agilent RNA 6000 Pico Kit.

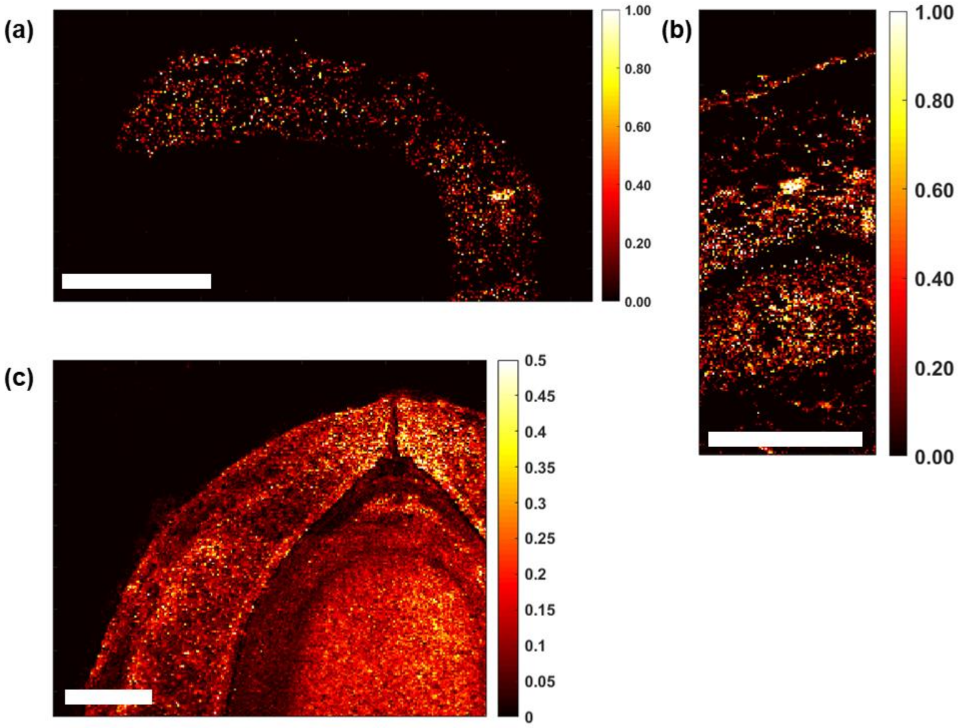

Fig. S3. Transverse distribution of phosphatidylcholine 34:2 [PC(34:2)] in biosynthetic tissues of narrow-leafed lupin (*L. angustifolius*) as determined by matrix-assisted laser desorption ionization mass spectrometry imaging (MALDI-MSI) at a spatial resolution of 5 µm. Each image was obtained by selecting the exact mass of PC(34:2) (± 5 ppm, Table S1) and normalizing by the total ion current (TIC). The color scale represents signal intensity. **(a)** Cross section of leaf. Bar = 0.4 mm. **(b)** Cross section of stem. Bar = 0.4 mm. **(c)** Cross section of pod. Bar = 1.0 mm.

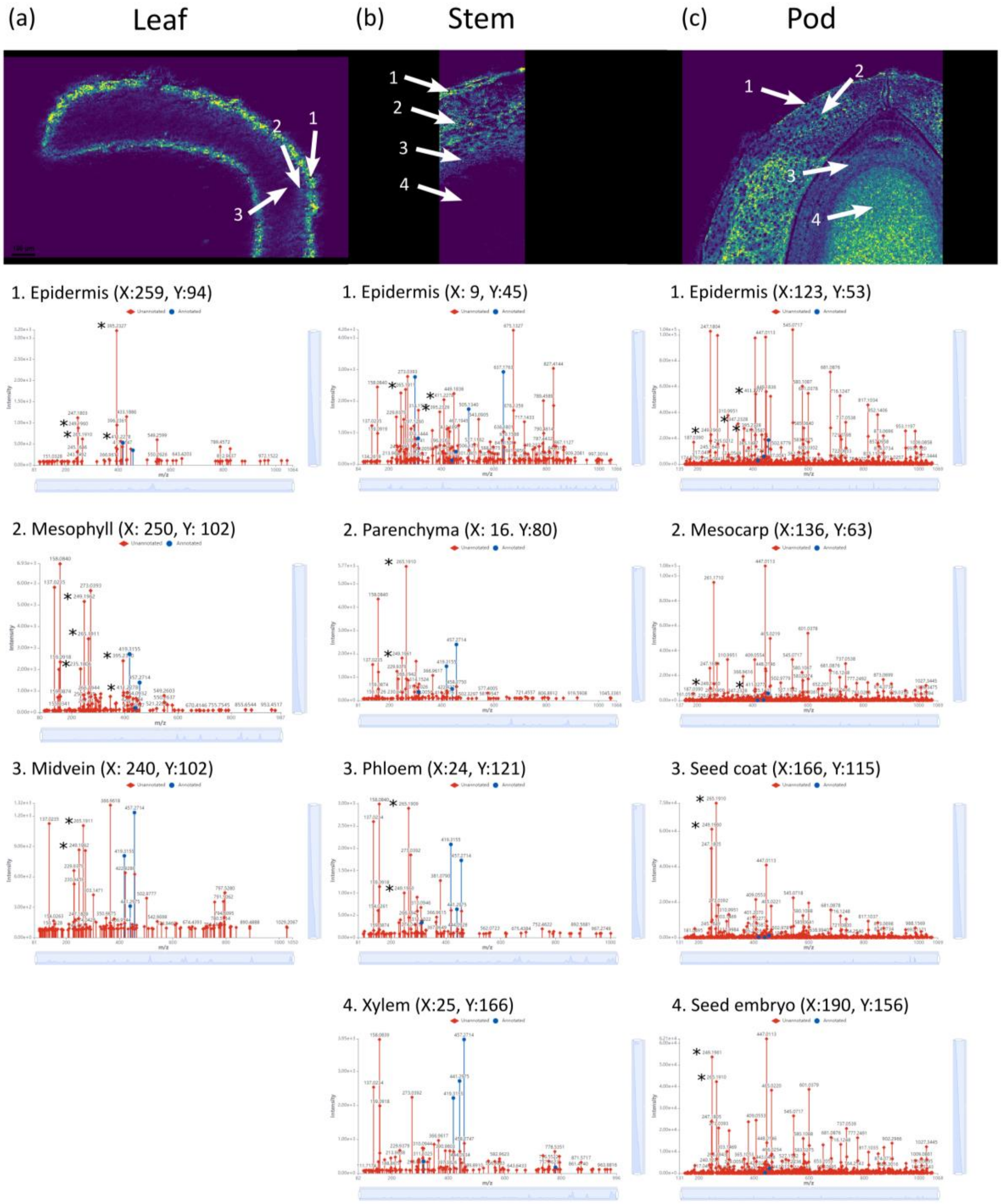

Fig. S4. Mass spectra of selected pixels within the narrow-leafed lupin (*L. angustifolius*) MALDI-MSI dataset. Pixels were selected from the indicated tissue types across **(a)** leaf, **(b)** stem, and **(c)** pod tissue sections. White arrows indicate the specific pixels for which the mass spectra are presented. Asterisks in the spectra highlight the *m/z* values associated with QAs (see list of expected *m/z* values in Table S1 above). The mass spectra can be explored further on METASPACE (https://metaspace2020.eu/).

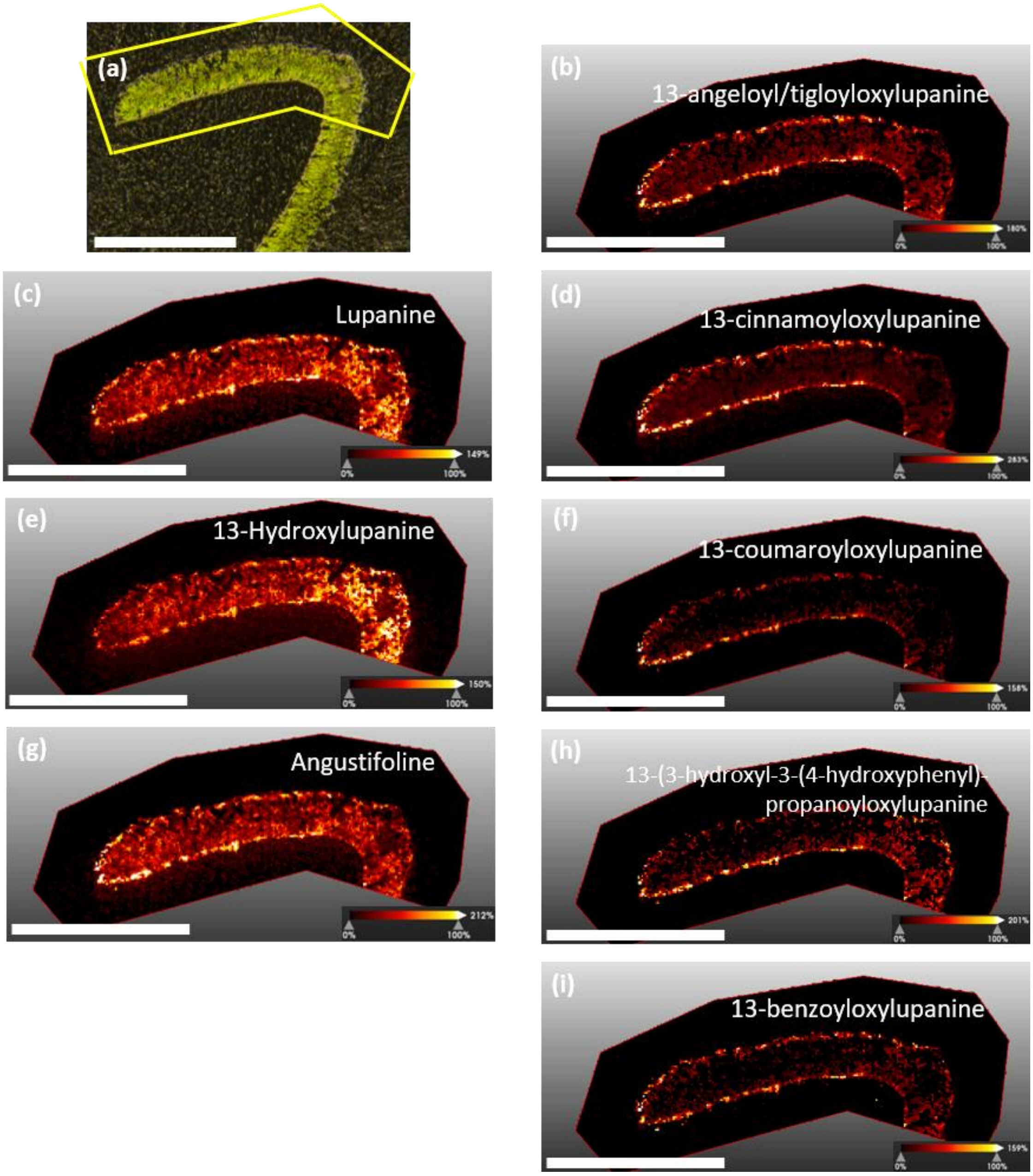

Fig. S5. Example of the replication of the results presented in Fig. 1. The figure shows the transverse distribution of quinolizidine alkaloids (QAs) in a leaf of narrow-leafed lupin (*L. angustifolius*) as determined by matrix-assisted laser desorption ionization mass spectrometry imaging (MALDI-MSI). **(a)** Bright-field microscopy image, with the yellow area denoting the approximate area that was further analyzed by high-res MALDI-MSI. **(b–i)** Individual MALDI-MS images of eight QAs at a spatial resolution of 10µm (bar = 1 mm). Each image was obtained by selecting the exact mass of the protonated QA (± 5 ppm, Table S1) and normalizing with the Root Mean Square (RMS) method. The color bars show intensities from 0 to 100%, with the maximum intensity indicated to the right (above 100%).

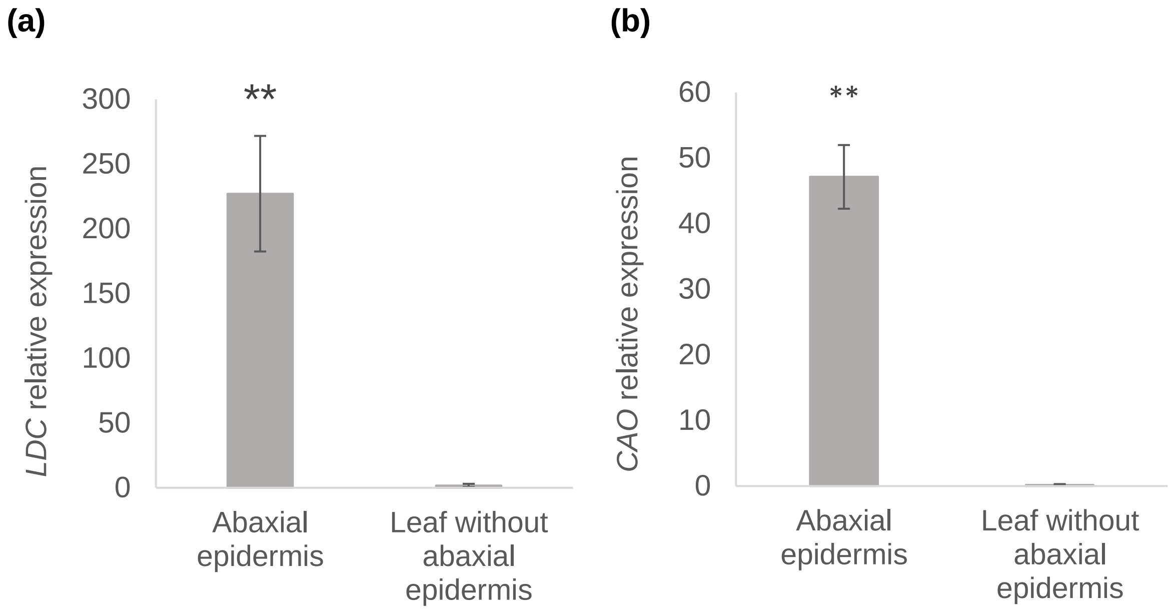

Fig. S6. Expression of two quinolizidine alkaloid biosynthetic genes in the leaf abaxial epidermis of narrow-leafed lupin (*L. angustifolius*) compared to the rest of the leaf without abaxial epidermis as determined by qPCR. Columns represent the mean of three biological replicates, and error bars represent the standard deviation. Asterisks indicate significant differences ($P < 0.001$), as determined by Student's t-test. **(a)** *LDC* expression**. (b)** *CAO* expression.

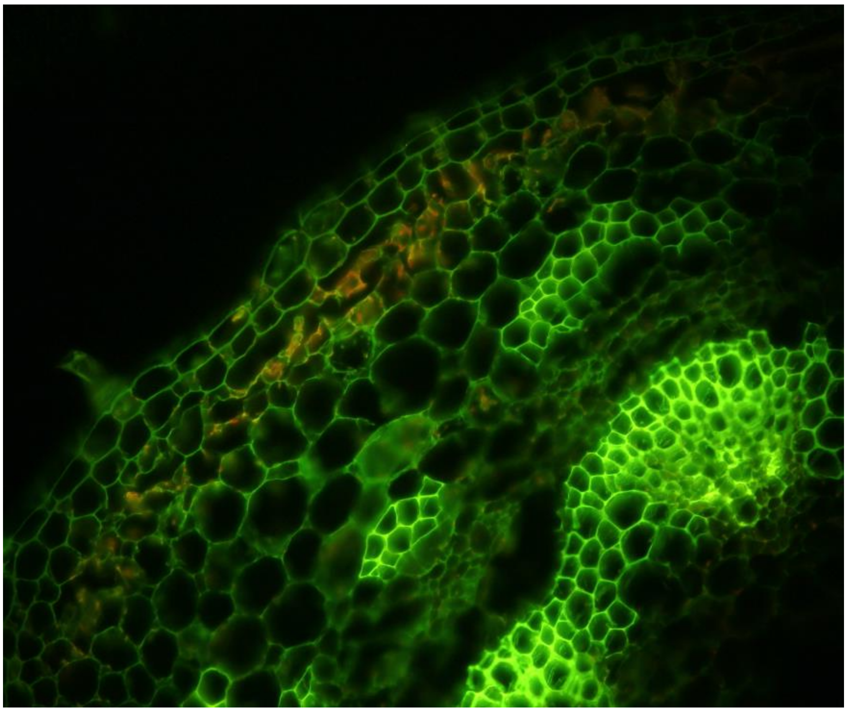

Fig. S7. Fluorescence microscopy image of a cross section of a stem of narrow-leafed lupin (*L. angustifolius*). Chlorophyll auto fluorescence (red) can be seen in the first 1-3 outer layers of the stem parenchyma. Excitation: band pass 450-490 nm; emission: long pass 515 nm.

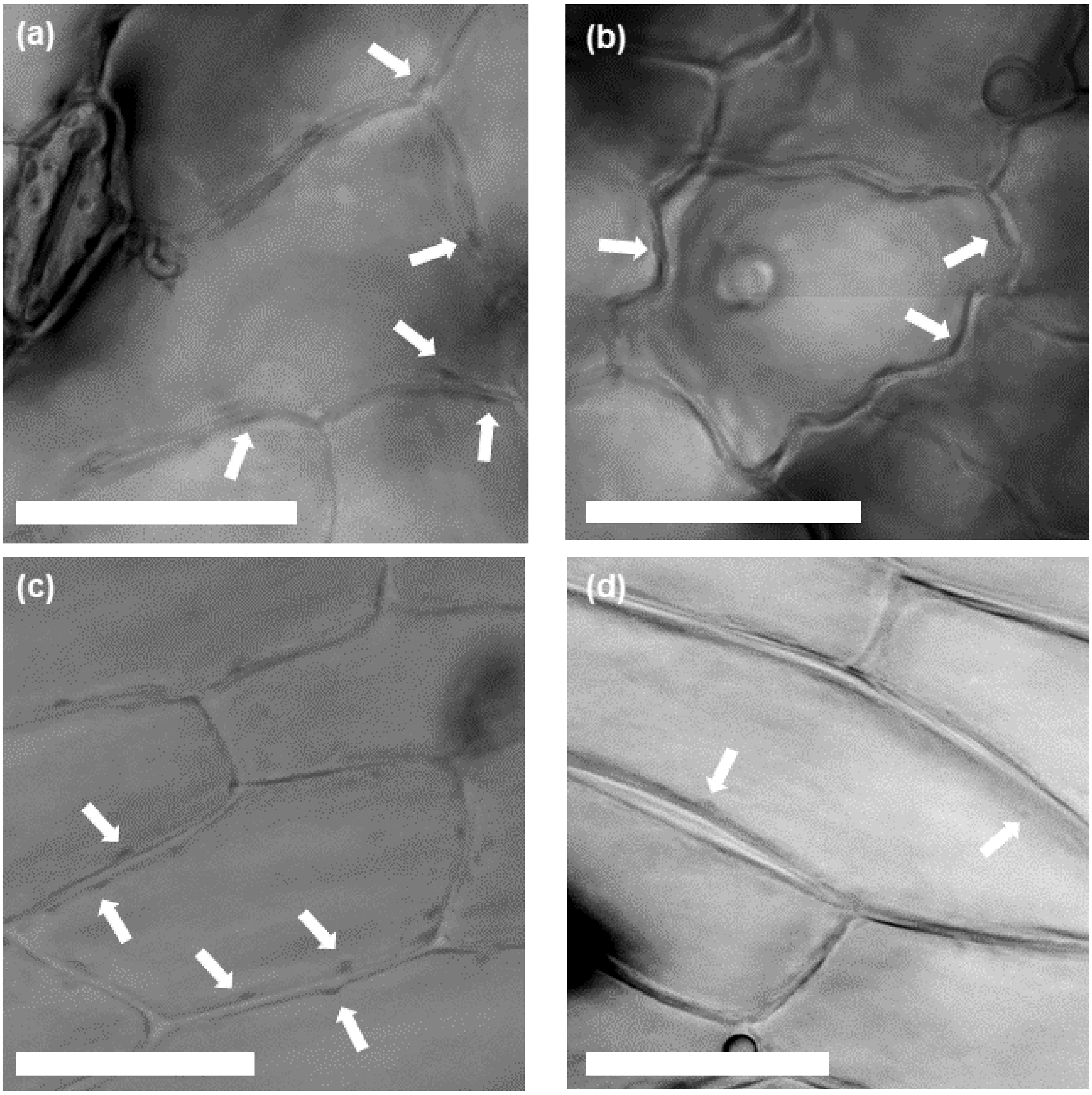

Fig. S8. Bright-field microscopy images of intact biosynthetic organs of narrow-leafed lupin (*L. angustifolius*). White arrows indicate some of the darker patches associated to the chlorophyll autofluorescence visualized in Fig. 5 and likely corresponding to chloroplasts (not all chloroplasts are indicated). Bar = 40 µm. **(a)** Leaf abaxial epidermis. **(b)** Leaf adaxial epidermis. **(c)** Stem epidermis. **(d)** Developing pod epidermis.

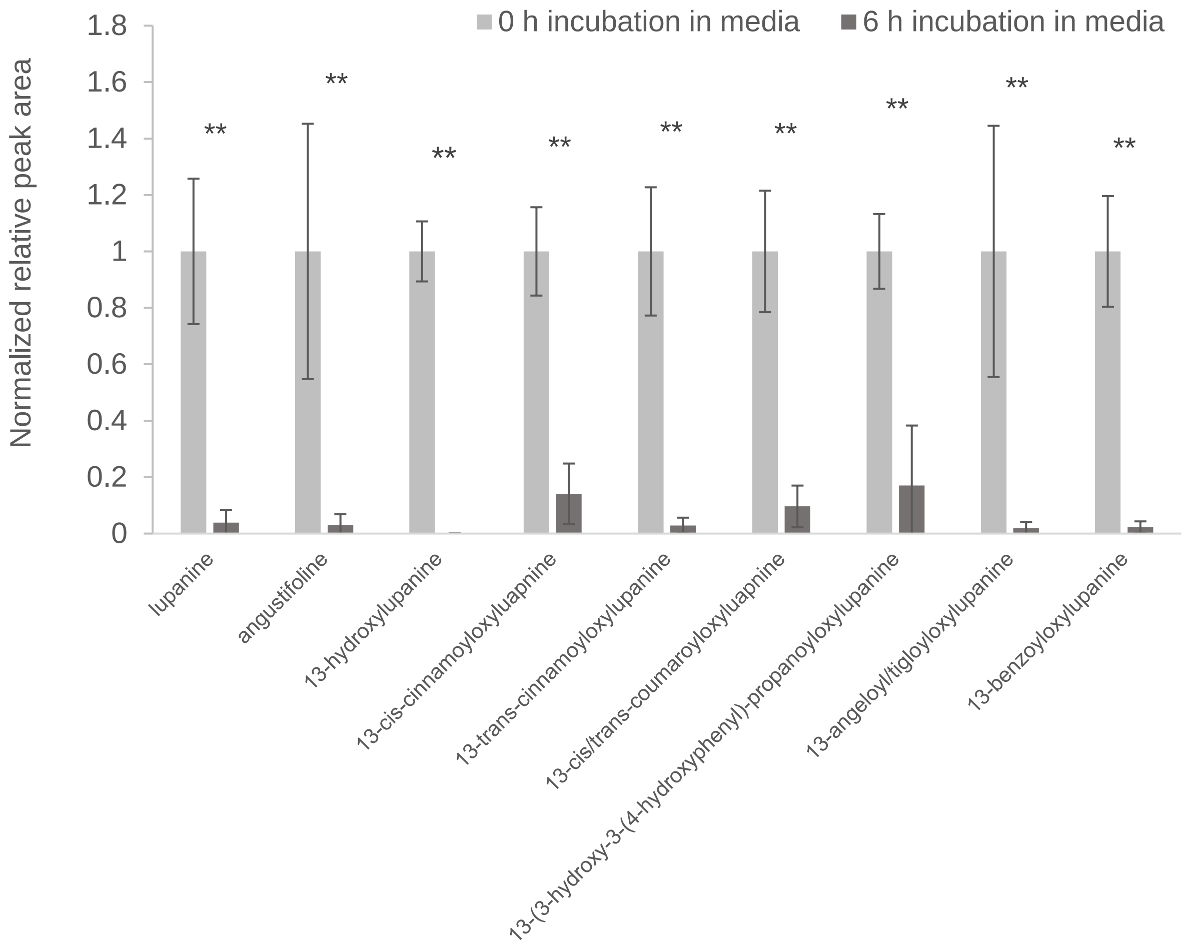

Fig. S9. Normalized relative peak area of unlabelled quinolizidine alkaloids (QAs) in leaf abaxial epidermis of narrow-leafed lupin (*L. angustifolius*) after 0 h and 6 h incubation in media (B5 medium, 2% sucrose and 1–5 mM labelled lysine) as determined by LC-MS. QAs were identified by their *m/z* values (Table S1). Peak areas were determined relative to that of caffeine (internal standard) and were normalized to the corresponding values at 0 h. Asterisks indicate significant differences ($P < 0.001$) between levels of individual QAs at 0 h and 6 h as determined by Student's t-test. Rectangular bars and error bars represent the mean and standard deviation of two (0 h) or four (6 h) biological replicates.

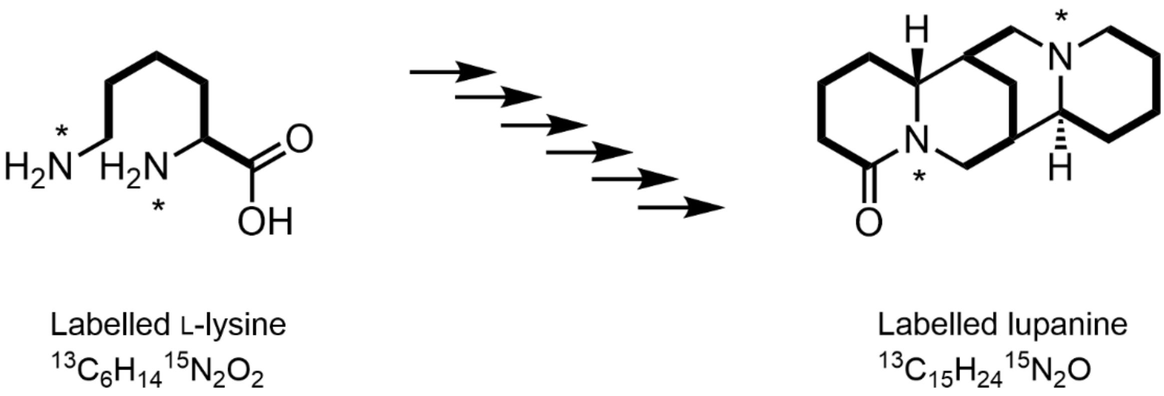

Fig. S10. Incorporation of the isotopically labelled L-lysine used in the feeding experiments into the tetracyclic quinolizidine alkaloid backbone (derived from three units of L-lysine). Lupanine is depicted as an example. The bonds in bold indicate the presence and incorporation of ^{13}C, and the asterisks indicate the presence and incorporation of ^{15}N. A compilation of previous precursor feeding experiments has been presented by Mancinotti *et al.* (2022).

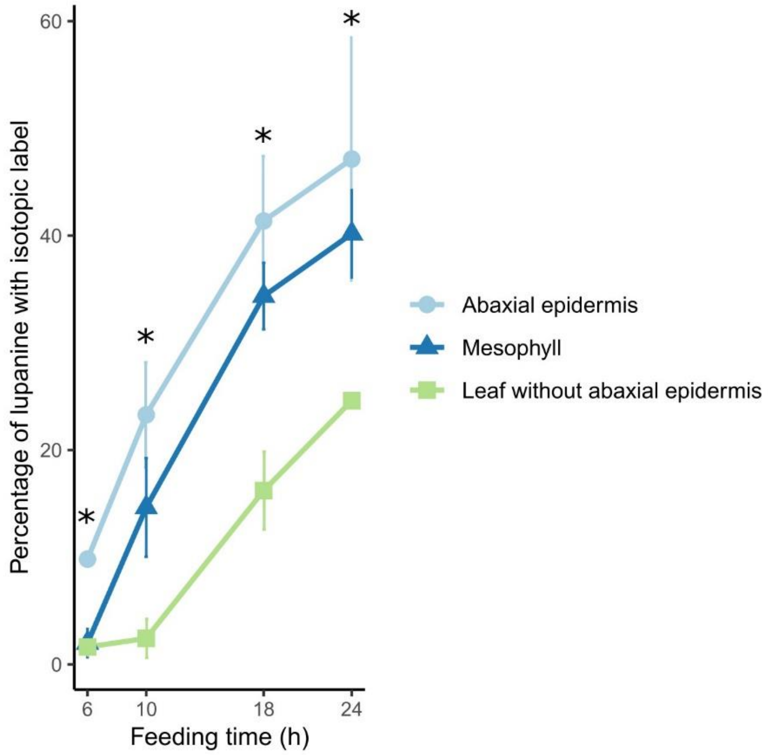

Fig. S11. Percentage of total amount of lupanine with isotopic label in tissue fractions of narrow-leafed lupin (*L. angustifolius*) leaves upon feeding with labelled L-lysine. Single asterisks represent either a significant difference between abaxial epidermis and the mesophyll (6 h), or a significant difference between abaxial epidermis and the remaining leaf tissue (10, 18, and 24 h) (*Padj* < 0.05 on Tukey's test). Data points and error bars represent the mean and standard deviation of 3 biological replicates.

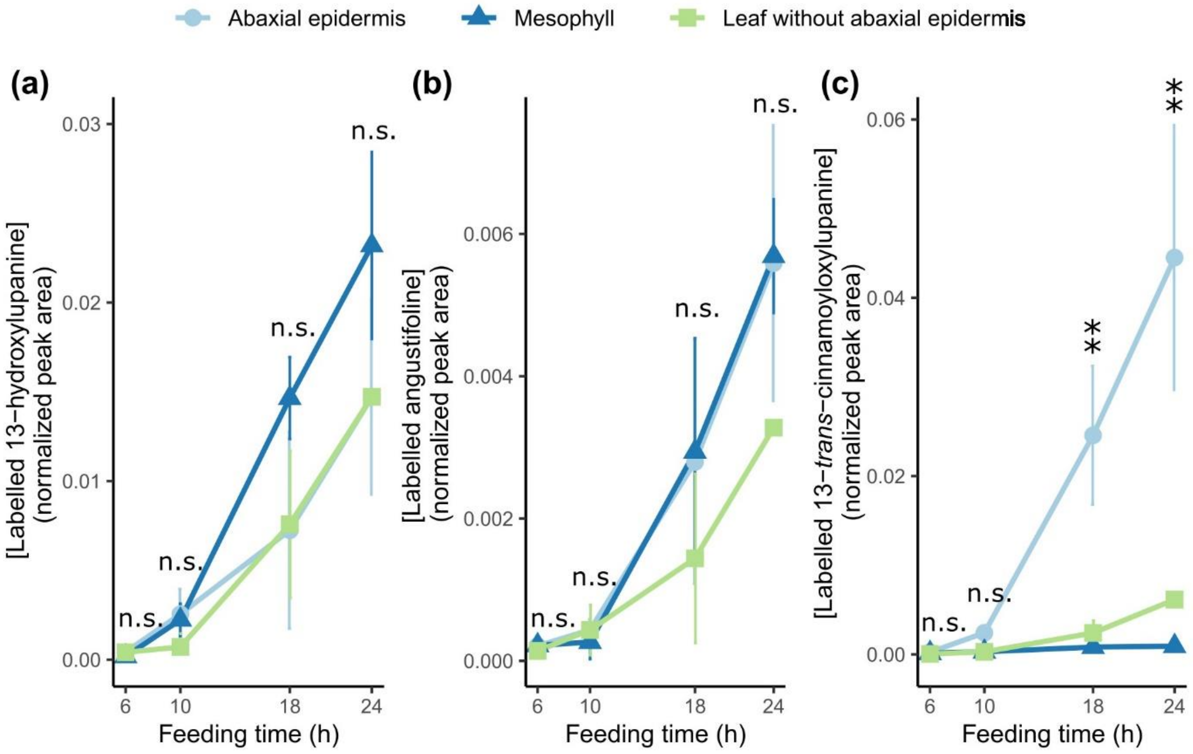

Fig. S12. Trace accumulation of three isotopically labelled quinolizidine alkaloids (QAs) in tissue fractions of narrow-leafed lupin (*L. angustifolius*) leaves upon feeding with labelled L-lysine. Whole leaves were fed via the cut petiole, and leaves were dissected at four time points after the start of the feeding period (6, 10, 18, and 24 h). The respective tissue fractions (abaxial epidermis, mesophyll, and leaf without abaxial epidermis) were analyzed by LC-MS. **(a)** Accumulation of the core QA 13-hydroxylupanine in the tissue fractions across time points. **(b)** Accumulation of the core QA angustifoline in the tissue fractions across time points. **(c)** Accumulation of the QA ester 13-*trans*-cinnamoyloxylupanine in the tissue fractions across time points. The double asterisks represent significant differences between abaxial epidermis and both the remaining leaf tissue and the mesophyll (*Padj* < 0.05 on Tukey's test). ns = not significant. Data points and error bars represent the mean and standard deviation of 3 biological replicates.

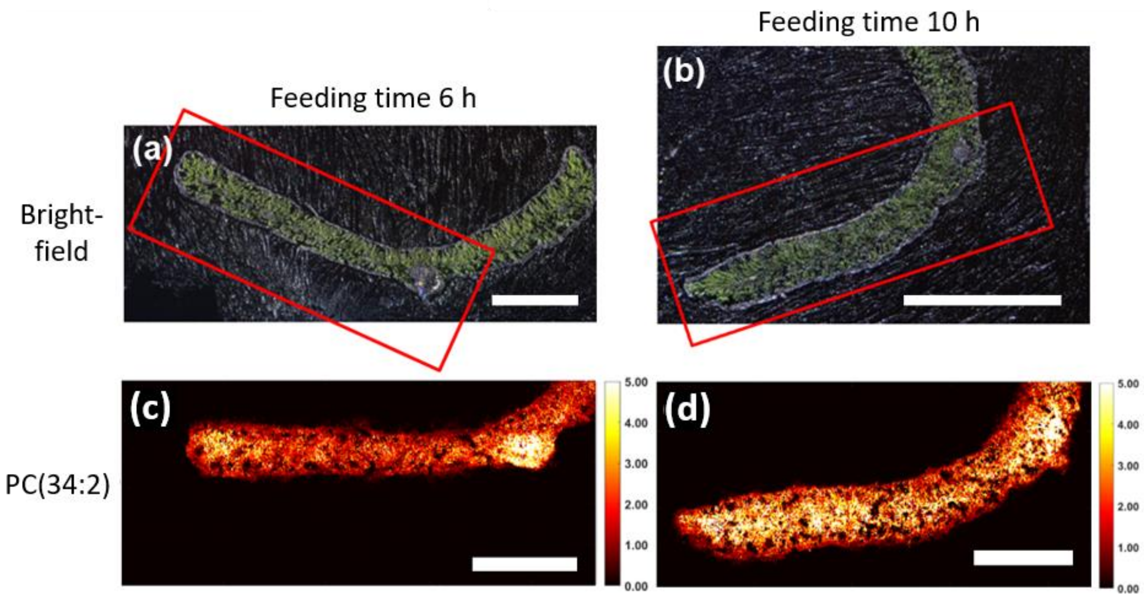

Fig. S13. Distribution of control membrane component phosphatidylcholine 34:2 [PC(34:2)] in narrow-leafed lupin (*L. angustifolius*) transverse leaf sections at 6 h and 10 h after feeding with isotopically labelled L-lysine. **(a,b)** Bright-field microscopy images with red box denoting the area that was analyzed by MALDI-MSI (bar = 1 mm). **(c–d)** MALDI-MS images of PC(34:2) at 5 µm spatial resolution (bar = 0.5 mm). Each image was obtained by selecting the exact mass of the potassium adduct of PC34:2 (± 5 ppm, Table S1) and normalizing by the total ion current.

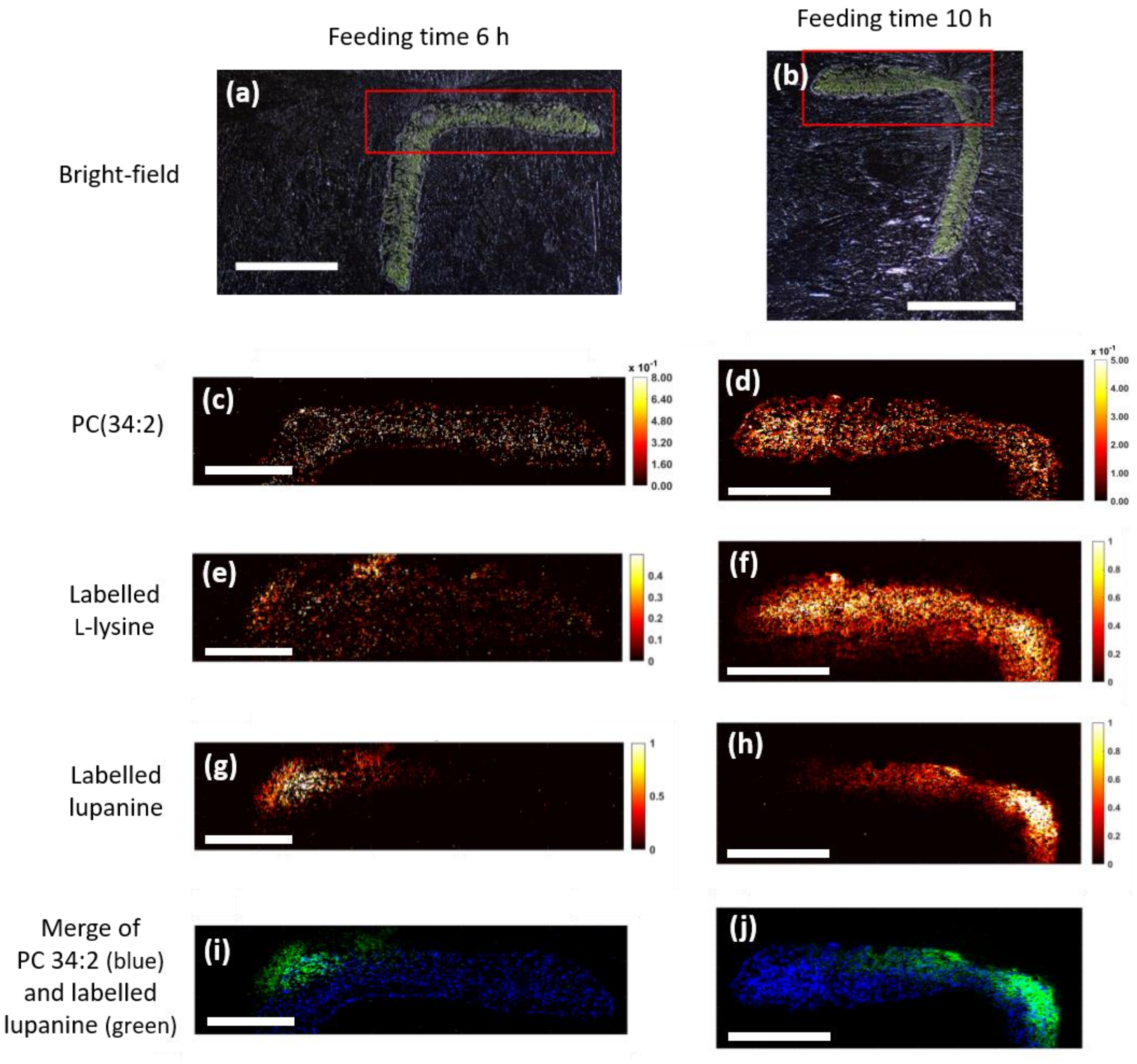

Fig. S14. Replicate of the experiment shown in Fig. 7 (MALDI-MS imaging of leaves fed with isotopically labelled L-lysine at the two first time points). The figure shows the distribution of phosphatidylcholine 34:2 [PC(34:2), control membrane component], isotopically labelled L-lysine and isotopically labelled lupanine in transverse leaf sections of narrow-leafed lupin (*L. angustifolius*) at 6 h and 10 h after feeding with isotopically labelled L-lysine. **(a,b)** Bright-field microscopy images with red box denoting the area that was analyzed (bar = 1 mm). **(c-h)** MALDI-MS images of PC(34:2), labelled L-lysine, or labelled lupanine at 5 µm spatial resolution (bar = 0.5 mm). Each image was obtained by selecting the exact mass of either the proton adducts (for labelled L-lysine or labelled lupanine) or the potassium adduct (for PC34:2) (± 5 ppm, Table S1) and normalizing by the total ion current. **(i,j)** Merged MALDI-MS images of PC(34:2) (blue) and labelled lupanine (green) (bar = 0.5 mm).

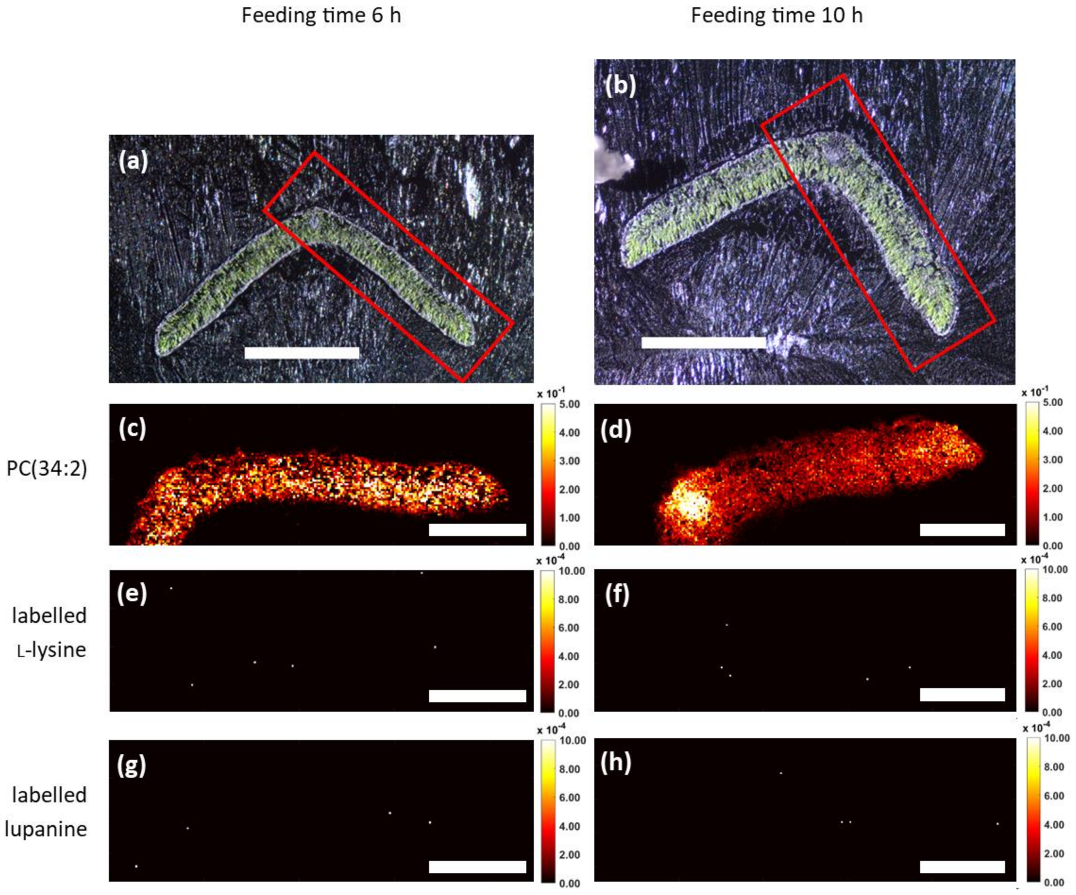

Fig. S15. Distribution of phosphatidylcholine 34:2 [PC(34:2), control membrane component] and labelled versions of L-lysine and lupanine (negative controls) in transverse sections of narrow-leafed lupin (*L. angustifolius*) leaves after feeding with unlabeled L-lysine. Images on the left correspond to leaves at 6 h after the start of the feeding. Images on the right show leaves at 10 h after the start of the feeding. All MALDI-MSI images were obtained at 10 µm spatial resolution. **(a,b)** Bright-field microscopy images, with red box denoting the areas that were further analyzed (bar = 1 mm). **(c–h)** MALDI-MS images of phosphatidylcholine (34:2), labelled L-lysine, or labelled lupanine at the indicated time points (bar = 0.5 mm). Each image was obtained by selecting the exact mass of either the proton adducts (for labelled L-lysine or labelled lupanine) or the potassium adduct (for PC34:2) (± 5 ppm, Table S1) and normalizing by the total ion current.

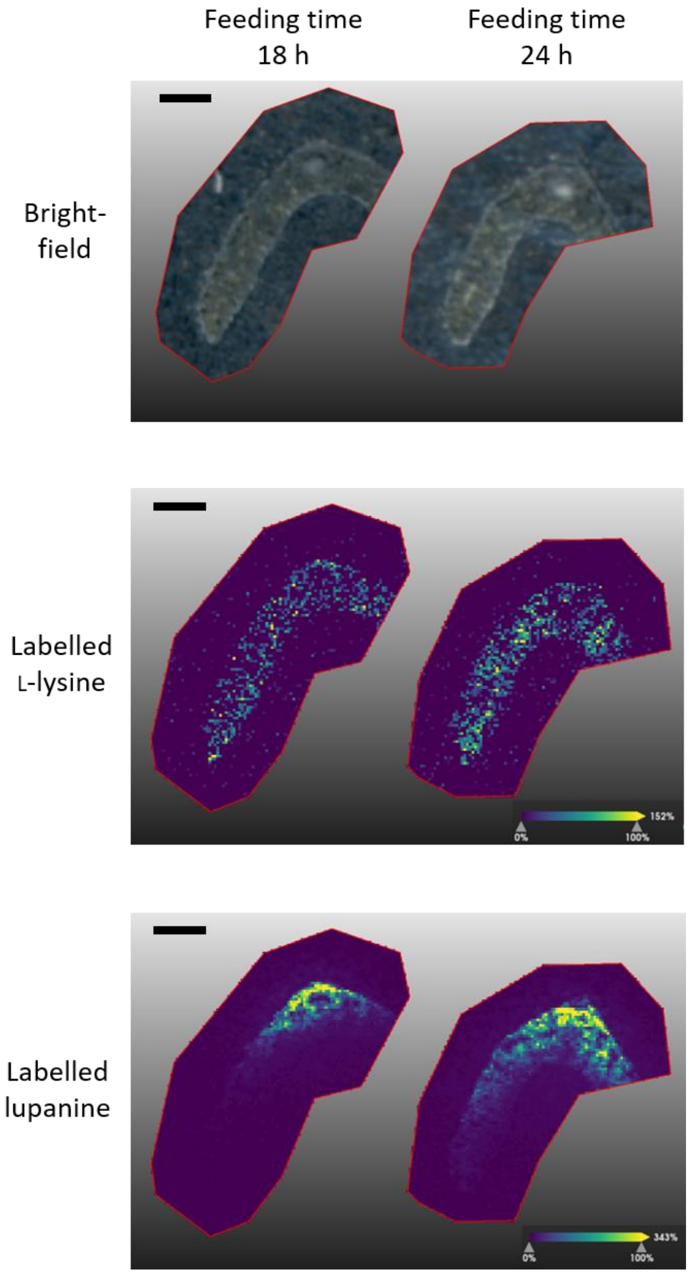

Fig. S16. Distribution of isotopically labelled L-lysine and isotopically labelled lupanine in transverse leaf sections of narrow-leafed lupin (*L. angustifolius*) at 18 h (images on the left) and 24 h (images on the right) after feeding with isotopically labelled L-lysine. The top panel corresponds to the bright-field microscopy images, whereas the middle and lower panels correspond to the MALDI-MS images of labelled L-lysine or labelled lupanine (respectively) at 10 µm spatial resolution (bar = 0.4 mm). Each image was obtained by selecting the exact mass of the target compound $[(M+H)^+]$ (± 5 ppm, Table S1) normalizing with the Root Mean Square (RMS) method. The color bars show intensities from 0 to 100%, with the maximum intensity indicated to the right (above 100%). The MALDI-MSI protocol used differed from the one used for imaging the early time points (6 h and 10 h). Thus, while the patterns and pattern progressions are informative, the intensities of the signals show here are not comparable to the images of the early time points (6 h and 10 h) shown in Fig. 7 & S14.

Video S1. Chlorophyll autofluorescence (red) in the narrow-leafed lupin (*L. angustifolius*) leaf abaxial epidermis and underlying cell layers (z-stack).

Video S2. Chlorophyll autofluorescence (red) in the narrow-leafed lupin (*L. angustifolius*) leaf adaxial epidermis and underlying cell layers (z-stack).

Video S3. Chlorophyll autofluorescence (red) in the narrow-leafed lupin (*L. angustifolius*) stem epidermis and underlying cell layers (z-stack).

Video S4. Chlorophyll autofluorescence (red) in the narrow-leafed lupin (*L. angustifolius*) developing pod epidermis and underlying cell layers (z-stack).

References

Mancinotti D, Rodriguez MC, Frick KM, Dueholm B, Jepsen DG, Agerbirk N, Geu-Flores F. 2021. Development and application of a virus-induced gene silencing protocol for the study of gene function in narrow-leafed lupin. *Plant Methods* **17:** 1-13.

Mancinotti D, Frick KM, Geu-Flores F. 2022. Biosynthesis of quinolizidine alkaloids in lupins: mechanistic considerations and prospects for pathway elucidation. *Natural Product Reports* **39:** 1423-1437.

Otterbach SL, Yang T, Kato L, Janfelt C, Geu-Flores F. 2019. Quinolizidine alkaloids are transported to seeds of bitter narrow-leafed lupin. *Journal of Experimental Botany* **70:** 5799-5808.
